# The new science of sleep: From cells to large-scale societies

**DOI:** 10.1371/journal.pbio.3002684

**Published:** 2024-07-08

**Authors:** Omer Sharon, Eti Ben Simon, Vyoma D. Shah, Tenzin Desel, Matthew P. Walker

**Affiliations:** 1 Department of Psychology, University of California, Berkeley, California, United States of America; 2 Helen Wills Neuroscience Institute, University of California, Berkeley, California, United States of America

## Abstract

In the past 20 years, more remarkable revelations about sleep and its varied functions have arguably been made than in the previous 200. Building on this swell of recent findings, this essay provides a broad sampling of selected research highlights across genetic, molecular, cellular, and physiological systems within the body, networks within the brain, and large-scale social dynamics. Based on this raft of exciting new discoveries, we have come to realize that sleep, in this moment of its evolution, is very much polyfunctional (rather than monofunctional), yet polyfunctional for reasons we had never previously considered. Moreover, these new polyfunctional insights powerfully reaffirm sleep as a critical biological, and thus health-sustaining, requisite. Indeed, perhaps the only thing more impressive than the unanticipated nature of these newly emerging sleep functions is their striking divergence, from operations of molecular mechanisms inside cells to entire group societal dynamics.

## Abstract 2

Sleep appears to be a universal state across the animal kingdom, suggesting it offers a conserved benefit to organismal health. This Essay highlights eight of the most exciting new discoveries within sleep science, discussing how these have expanded our understanding of sleep’s function at the cellular, organismal, and societal levels.

## Introduction

Sleep appears to be a universal, highly conserved state across the animal kingdom [1,2]. This fact would perhaps suggest a common single function of sleep that transcends phylogeny; however, proving this has been far more challenging than anticipated. Indeed, science has struggled to answer, with universal agreement, the basic question of why it is that we sleep and, to an ever greater degree in humans, why we dream. Yet, in the past 2 decades, more has arguably been uncovered about the polyfunctional nature of sleep than in the previous 200 years. Building on a wave of exciting recent discoveries, in this Essay, we provide a select collection of highlights from sleep research across genetic, molecular, cellular, whole body, whole brain, group-social, and societal levels.

This Essay is not meant to serve as a comprehensive review of sleep research nor an exhaustive cataloging of all recent discoveries. Rather, we aim to provide the reader with a sampling of representative new research areas. Towards that end, the Essay is structured into several main sections that traverse a descriptive narrative, from cells to society, each exploring different facets of sleep science. We start with recent discoveries at the level of DNA and genes, describing both genes that control sleep duration, and the newly revealed role of sleep in DNA repair. Thereafter, we ascend to physiological systems, one example of which focuses on very recent findings regarding an intimate and bidirectional link between sleep and the gut microbiome. Next, we address exciting recent work seeking to develop new technologies to augment and enhance human sleep, ranging from electrical to acoustic, kinesthetic, and thermal manipulations, all of which have marked therapeutic and intervention implications.

Having considered sleep functions within the body, we then move higher into the brain. We address novel sleep functions at the neural level, including sleep’s role in regulating the glymphatic cleansing system. Staying within the brain, we then address one of the newest emerging fields of sleep neuroscience, that of emotional wellness and mental health. Here, the latest findings move beyond sleep’s role in basic emotional regulation and, instead, signal a clear and intimate connection between sleep and complex socioemotional functions within an individual, between individuals, across large groups of individuals, and across entire societies.

Staying with the theme of sleep across groups, we then investigate sleep’s evolutionary roots across phylogenetic groups, providing a very different approach to understanding the functions of sleep. We describe new work that seeks to explain the vast, previously perplexing, and impressively large differences in sleep quantity and physiological sleep quality (Glossary) across species. From such an examination come powerful insights into the universal function(s) of sleep that only this type of approach can reveal. Finally, we move past the basic physiological state of sleep into the altered psychological state of human consciousness called dreaming. We outline both prior and the latest evidence regarding the functional importance of dreaming in service of memory enhancement, creativity, and emotional first aid, independent of the rapid eye movement (REM) sleep (Glossary) state such dreams emerge from.

**Glossary**

**Sleep quality**

Evaluated through both subjective means, which involves individual’s self-reporting their perceived quality of sleep, and objective methods, including measurements of sleep stages or quantitative electroencephalography brainwave metrics.

**Rapid eye movement (REM) sleep**

Also referred to as paradoxical sleep, REM represents a sleep phase marked by a desynchronized electroencephalogram with high-frequency, low-amplitude activity (especially in the theta band), rapid movement of the eyes, muscle immobilization, and the occurrence of dreams.

**Nonrapid eye movement (NREM) sleep**

Describes the sleep phase that encompasses the period between falling asleep and reaching deep sleep, yet is not REM sleep. The stages of NREM sleep are typically categorized into 3 categories: N1 (shallow sleep), N2 (light sleep), and N3 (deep sleep).

**Slow-wave activity (SWA)**

A characteristic electrophysiological pattern marked by slow, synchronized oscillations in the 0.5 to 4.0 Hz range. SWA reaches its peak during NREM sleep and diminishes across the night, reflecting the discharge of homeostatic sleep pressure that builds the longer an individual is awake.

**Obstructive sleep apnea**

A sleep disorder characterized by recurrent impaired or absent breathing during sleep, as well as by reductions in blood oxygen saturation, caused by airway occlusion.

**Sleep restriction**

A decrease (but not total absence) of sleep across the prior night or nights. Amounts typically range from 1 to 6 hours of sleep reduction. Sleep restriction is often termed chronic if it persists for more than 24 hours.

**Gut dysbiosis**

An imbalance in the gut’s microbial community, potentially leading to health issues. It involves a decrease in beneficial bacteria and an increase in harmful ones, disrupting normal gut function. Restoring balance is crucial for overall health.

**Disrupted sleep**

Irregular sleep patterns characterized by insufficient sleep duration, disrupted sleep cycles (such as altered sleep architecture), and/or reduced sleep quality (evaluated through measures like spectral electroencephalogram power).

**Allostatic distress**

A state reflecting the cumulative physiological damage caused by chronic stress, in part stemming from prolonged activation of stress-related messengers like cortisol and adrenaline. Allostatic distress is associated with disruption of adaptive biological systems and responses, including those related to the hypothalamic-pituitary-adrenal (HPA) axis and immune function, ultimately contributing to various health issues.

**Cognitive behavioral therapy for insomnia**

A scientifically supported approach to treating insomnia that involves a comprehensive psychological intervention aimed at addressing the underlying behaviors and thought patterns associated with insomnia.

**Functional connectivity**

Within functional MRI, the statistical association observed between activity signals originating from 2 or more anatomically separate brain regions.

Through these exciting new discoveries, and many others like them, we have come to recognize that sleep has evolved to support polyfunctional processes for the brain and body. Moreover, such powerful new evidence reaffirms sleep as a biologically critical and health-sustaining requisite—a requisite for reasons: that are surprising in their nature.

## Genes linked to short sleep need

**Fig 1:**
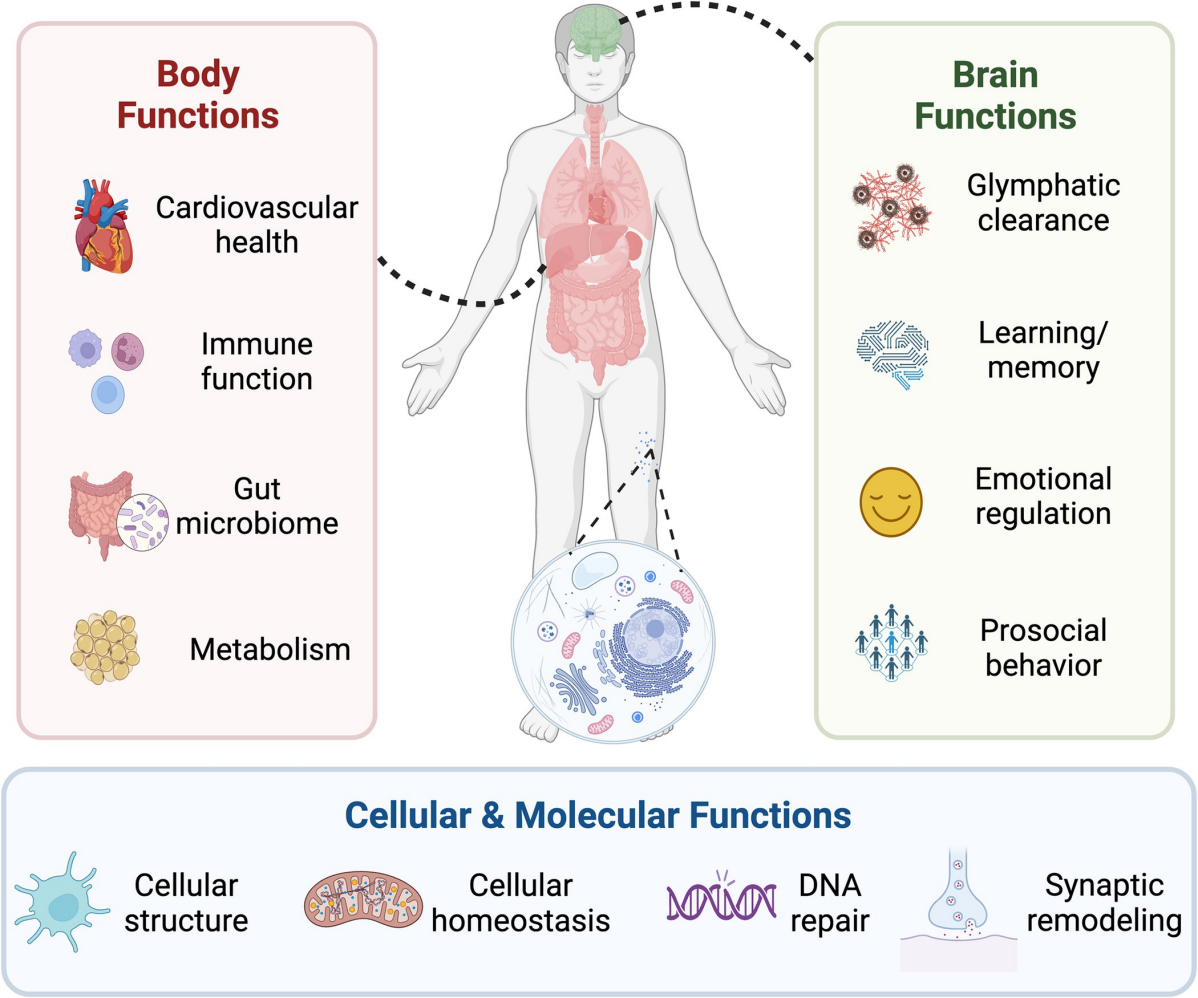
The necessity of sleep at multiple levels. Sleep serves a multitude of functions for humans. These functions exist at multiple physiological levels, from cells (bottom panel) to bodily systems (left panel), through to multiple brain functions and systems (right panel).

Insufficient sleep exacts a significant toll on all cognitive and emotional brain functions and impacts all major physiological systems of the body, from the immune, cardiovascular, thermoregulatory, metabolic, and reproductive systems, to respiratory and endocrine systems (Fig 1). Unsurprisingly, then, insufficient sleep also predicts all-cause mortality risk [1,3,4]. Nevertheless, there is a common claim by some that, “I’m one of those individuals who can function just fine on 5 hours of sleep or less.” While this is unlikely, based on the extent of empirical findings [5–8], a select collection of individuals do seem to be exceptions to the recommended 7- to 9-hour sleep requirement, on the basis of gene mutations that reduce sleep need [5]. Termed “natural short sleepers,” this small set of individuals appears to have a natural sleep requisite as low as 6 to 6.25 hours per night without showing any observable cognitive deficits assessed so far [5].

The first genetic variant accounting for these natural short sleepers centered on a variation in *DEC2* gene identified in families of naturally short-sleeping humans. Initial work focused on dizygotic twins, each of whom differed on the basis of this *DEC2* gene variant (standard versus mutation). The twins had their daily sleep–wake patterns measured and also came to the sleep laboratory for full sleep physiological recordings. The data revealed that the twin with the *DEC2* mutation naturally slept 30 to 60 minutes less than their noncarrier twin in both real-world and laboratory-assessed environments [9].

This was not the most interesting result, however. Following a 38-hour sleep deprivation period, the twin carrying the short-sleeping genetic variant exhibited greater resilience to sleep deprivation, defined by performance on select cognitive tasks, having only half the number of attentional failures compared to their noncarrier sibling. The final revelation emerged during the subsequent night of recovery sleep. Typically, following sleep loss, individuals sleep notably longer, indicating a buildup of sleep debt that is proportional to the amount of extended time awake. The longer the prior waking period, the longer and deeper the recovery sleep. However, the *DEC2* mutant carrier did not show this normal strong sleep–rebound response, obtaining 1.5 hours less recovery sleep than their noncarrier twin. This finding once again indicates a reduced innate sleep need, here even under the pressure of prior sleep deprivation. Similar results have been observed in *DEC2* mutant mice [5], with wild-type mice showing a 70% increase in NREM sleep (Glossary) following sleep deprivation, compared to a 17% increase in the *DEC2* mutant mice.

### How is short sleep achieved?

Using mice genetically engineered to carry the short-sleeping genes identified in humans, new findings have revealed how and why the *DEC2* mutation may afford a reduced sleep need [7,8]. The *DEC2* mutation results in an increased expression of the wake-promoting neurochemical, orexin [10]; i.e., natural short sleepers with the *DEC2* variant have an amplified neurochemical wake–drive, resulting in prolonged wakefulness across the day and thus shorter sleep duration at night. However, this insight does not wholly explain the reduced homeostatic sleep need after sleep deprivation in individuals carrying the *DEC2* mutation. If anything, a strong drive for wakefulness may be predicted to result in a stronger buildup of sleep homeostatic factors, such as adenosine, that would increase homeostatic sleep needs during postdeprivation recovery.

An additional explanation for the innate reduced general sleep amount (approximately 6 hours), and one that may account for a reduced homeostatic sleep need, concerns the electrical efficiency of deep-sleep brain waves. A physiological measure of deep NREM sleep quality is slow electrical brainwave activity, also known as slow-wave activity (SWA, <4 Hz; Glossary). In the aforementioned *DEC2* twin studies, across all 3 nights recorded in the laboratory, the short-sleeping twin exhibited significantly greater SWA, one interpretation of which is that their deep NREM sleep was of superior electrical quality. By means of this superior SWA power, short-sleeping individuals may be able to dissipate the accumulation of sleep needs based on time awake during the day, and in doing so, reduce the total amount of time needed for sleep [9], thus increasing sleep efficiency and decreasing sleep need.

Another mutation has also been discovered in natural short sleepers. *ADRB1* gene governs the beta-1 adrenergic receptor, which influences sleep–wake regulation. Much like the *DEC2* mutation, those carrying the *ADRB1* mutation display increased SWA during NREM sleep early in the night. Moreover, the speed of decline in SWA across the night—potentially reflecting a more efficient evacuation of accumulated sleep pressure across the waking day—was faster in those carrying this mutation. Again, this points to the possibility of superior deep-sleep electrical brainwave activity, increasing sleep efficiency and, hence, decreasing the amount of sleep needed [6].

This emerging picture of superior deep-sleep physiology in short sleepers is not, however, exclusive to NREM sleep. In several short-sleeping studies, alterations in REM sleep have also been identified, the reasons and function(s) of which are more mysterious; e.g., in the dizygotic *DEC2* twin studies, following a sleep deprivation phase, the noncarrier twin spent nearly 2 additional hours in REM sleep during recovery sleep, as is typical. However, the carrier twin showed almost no change in the rebound of REM sleep [9], indicative of a reduced REM sleep need as well. Similarly, wild-type mice exhibit a 175% increase in REM sleep after sleep deprivation, yet short-sleeping *DEC2* mutant mice expressed only a 74% relative increase in REM sleep [5]. *ADRB1* short-sleeping mutant mice similarly do not show the same REM sleep need relative to wild-type mice under normal (nondeprived) sleeping conditions [6]. Short-sleeping gene variants, therefore, seem to require less total sleep, but also less REM sleep. There are still no clear answers as to why.

### Is short sleep without true cost?

Arguably the most fundamental question in the emerging description of short sleep is that of cost—is there truly no health cost to these short-sleeping individuals? Cross-sectional analyses suggest that cognitive functions do not suffer, relative to controls without the DEC2 genetic variant, yet there have been no systematic studies assessing other known sleep-dependent brain and body functions (Fig 1). Furthermore, no prospective longitudinal studies of natural short sleepers have been conducted to determine whether the health span and/or life span are similar to controls, or for twins relative to their noncarrier sibling. An assumption of no true cost, therefore, remains a hopeful one, but an assumption nevertheless. One relevant example that may temper optimism concerns work in fruit flies using the “Shaker” gene mutation that shortens sleep duration [11]. Evaluated longitudinally, the life span of these mutant flies was significantly shorter relative to wild-type flies. This would suggest that some short sleep gene variants, at least in certain species, may come with a consequence only when assessed longitudinally, in this instance, premature mortality.

Genes not only affect sleep, but the reverse is also true. During time spent awake, the double-stranded backbone of DNA accumulates breaks. This damage is specific to neurons compared with nonneural brain cells such as Schwan cells or peripheral endothelial cells [12]. However, during sleep, these double-strand breaks are repaired rapidly [13], suggesting that a lack of sleep can induce excessive mutations and potentially explain why sleep is so evolutionary conserved. These findings also support the view that sleep is especially critical for the brain with regard to the cellular function of neurons, although it is possible that neural cells in the periphery (e.g., in the enteric system) are similarly affected. Fascinatingly, the DNA damage response, in turn, can impact sleep: Expression of the DNA repair enzyme PARP1 can induce sleep [14].

## The gut microbiome: A sleep interface?

Sleep, it was logically believed, primarily serves the sleeping organism itself. This view has changed, or at least been revised, in a model of symbiosis. Within us lives a diverse community of microorganisms, particularly in our gut, collectively known as the gut microbiota. The gut microbiota is composed of several billion bacteria, viruses, fungi, and additional microbes [15,16] and is known to influence a broad swathe of host physiology and behavior [17]. Dysfunction of the microbiome is now linked to numerous disorders and conditions, including obesity, type 2 diabetes, cardiometabolic diseases, nonalcoholic liver disease, and several immune disorders, as well as neurological disorders such as autism spectrum disorder, Alzheimer’s disease, depression, multiple sclerosis, Parkinson’s disease, and stroke [18–20]. Seminal work by Toth and Krueger [21,22] first linked sleep and the microbiota in the 1980s. Although, this field of research is still in its embryonic stages, a plethora of recent work is now providing exciting, and many surprising, new insights to add to those made by Toth and Krueger many decades ago. This is of particular interest as it could further promote the way we think about the established link between sleep and immunity [23,24], as the microbiome is fundamental for the development, training, and operation of the host’s immune system [25,26]. Most alluring, this relationship between the microbiota and sleep is bidirectional, opening up the possibility that modifying the gut microbiota may be a new tool for improving human sleep.

### How sleep impacts the gut microbiota

Chronic sleep disruption alters the configuration of the gut microbiota in several deleterious ways. A pioneering study in mice investigated the effects of 4 weeks of repeated sleep interruptions. The mice were gently handled every 2 minutes to trigger awakening, mimicking the frequency of interruptions as a model of obstructive sleep apnea (Glossary) in humans [27]. After 9 days, the amount of Firmicutes bacteria in the gut, which are associated with the fermentation processes involved in energy extraction, increased. Conversely, Bacteroidetes species decreased, which is notable as they serve anti-inflammatory functions. As predicted, the mice had increased markers of inflammation and infection, including the number of macrophages and neutrophils. In tandem with these microbiota changes caused by a lack of sleep came an increase in food intake [27]. This resulted in escalating amounts of visceral fat, even though total body weight remained constant [27,28], suggesting an impact on how the body partitions energy when sleep loss alters the microbiome. Encouragingly, these changes subsided within 2 weeks of restoring healthy sleep.

Similar causal evidence in humans has since emerged, although with some inconsistencies. Two consecutive nights of sleep restriction (approximately 4 hours per night; Glossary) moderately increased the ratio of Firmicutes to Bacteroidetes in humans [29], similar to the results observed in the mice [27]. By contrast, in a study that looked at 1 week of similar 4-hour per night sleep restriction, the authors failed to detect a change in microbiota composition [30]. Increasing the severity of sleep restriction to 2 hours each night for 3 consecutive nights did, however, significantly reduce the diversity of microbiota in the gut, leading to dysbiosis (an imbalance in the microbial communities living in the gastrointestinal tract; Glossary). This was especially true for a decrease in Ruminococcaceae, which normally contributes to the production of short-chain fatty acids (e.g., butyrate) [31]. Short-chain fatty acids help improve gut outer barrier integrity and metabolism and regulate immune function and blood pressure [32]. Yet, the changes in the diversity of the microbiome were not accompanied by changes in gut permeability, at least when assessed using urine samples [31].

While most of society’s sleep debt is brought about by sleep restriction, there are circumstances in which total sleep deprivation is common and necessary, including in medicine, and for those working as emergency responders, in the military, in aviation, and in law enforcement. When individuals are acutely sleep-deprived for 40 hours [33], a dose-dependent escalation of gut dysbiosis unfolds, the severity of which increases the longer without sleep an individual goes. Replicating earlier studies in mice, the progressive dysbiosis is paralleled by increases in circulating inflammatory markers, including the pro-inflammatory cytokines IL-1, IL-6, and TNFα. In addition to securing sufficient sleep, new findings point to sleep regularity as an independent emerging factor in protecting gut health [34]. In experiments in rats, circadian rhythm disturbances triggered by an 8-hour circadian shift every 3 days can lead to imbalances in gut microbiota composition and rhythms [35]. In humans, greater objectively measured night-to-night variability in sleep duration, together with increased time awake after sleep onset and lower sleep efficiency, are associated with lower microbiome diversity [36]. Thus, irregular sleep patterns, especially if coupled with poor-quality sleep, interfere with stable profiles of gut microbiota, one consequence of which is poor metabolic health [37].

A clever study has added new insight into the link between dysbiosis and inflammation caused by insufficient sleep by using a combination of species [33]. If the microbiota of sleep-deprived humans is transplanted into well-rested, non-sleep-deprived mice, those mice experienced a significant increase in inflammation relative to mice who received a transplant from well-rested humans. In addition, these pro-inflammatory effects caused by lack of sleep extended into the brain, with levels of pro-inflammatory cytokines IL-1 and IL-6 increasing in the medial prefrontal cortex and dorsal hippocampus, while levels of the anti-inflammatory cytokine IL-10 decreased. These findings confirm at least one of the directions of effect, such that changes in the gut microbiota caused by a lack of sleep represent an explanatory path leading to systemic inflammation [33]. In addition to changes in circulating markers of inflammation, there was increased expression of Iba-1protein, an index of microglia activity (the brain’s primary immune cells) in the medial frontal cortex and hippocampus following transplantation. These findings suggest that the cognitive effects of sleep deprivation could, in part, be mediated by brain inflammation caused by the sleep-loss-induced changes in gut microbiota composition. It also provides a possible biological mechanism—changes in glial inflammatory activity—that might explain how and why chronic gut dysbiosis and brain disorders are related.

### How the microbiota impacts sleep

Like so many other core physiological consequences, the idea that a lack of sleep impairs the microbiome is perhaps to be expected, but the idea that the microbiome could conversely impact sleep is more novel. The first pioneering investigation into this topic involved a 4-week antibiotic regimen in mice to deplete their gut microbiota [38]. Following the antibiotic course, the mice experienced a 100,000-fold reduction in gut bacteria. However, the causal manipulation of the microbiome led to a significant impairment in their brain’s ability to generate normative sleep in several ways. First, the mice aberrantly flip-flopped back and forth between NREM and REM sleep, indicative of unstable sleep-state regulation. The wake phase also suffered after the microbiome had been depleted. The mice could not sustain robust wakefulness across this period, experiencing excessive wake-time sleepiness. Added to this were uncharacteristic intrusions of NREM and REM sleep during the wake phase when the mice should otherwise be alert; the latter stage also pervaded into the sleep phase. Even the electrical brainwave quality of REM sleep was abnormally slowed in the microbiome-depleted mice. While preliminary, and despite the potential impact of antibiotics treatment on sleep patterns, these findings offer promising therapeutic potential. If microbiota composition can alter sleep, microbiome-specific interventions to restore and improve sleep may be possible.

### The microbiota, sleep, and disease

Given that experimental sleep loss impairs the gut microbiota, disorders showing sleep disruption would be expected to show co-occurring impairments in the composition of the microbiota. Insomnia is one such confirmatory example. Both acute (lasting days to weeks) and chronic insomnia (lasting months to years) have now been linked to significant gut dysbiosis and a decrease in bacteria that produce short-chain fatty acids. Indeed, individuals with these conditions showed increases in circulating levels of the pro-inflammatory cytokine IL-1β, suggesting the increases in inflammatory response observed in sleep disruption in the lab are the everyday reality of individuals with insomnia [39]. Collectively, these cross-sectional observations reinforce experimental data indicating that disrupted sleep (Glossary) robustly compromises the gut microbiome.

Longitudinal studies tracking several hundred patients over a 6-year period have since found similar impairments. No matter whether patients were recently diagnosed with insomnia or had been experiencing insomnia for many months or years, all went on to show gut dysbiosis, relative to healthy individuals who slept well [31,40]. This included a reduction in Ruminococcaceae bacteria, notable for their varied functions, including regulating the gut barrier integrity that normally shields an organism from pathogens. Notably, patients who went on to recover from their insomnia ultimately became indistinguishable from healthy individuals in their microbiome composition.

### Possible mechanisms

Since sleep impacts the microbiome, and the microbiome alters sleep, how do these distant systems converse? We would tender several candidates. First, a lack of sleep skews eating behavior, increasing food intake, biasing preference for higher caloric foods, and driving up consumption of simple and complex carbohydrates [41]. This altered eating behavior could, by itself, alter the gut microbiota by increasing the level of energy-extracting bacteria, which are responsible for digesting 10% to 30% of the nutrients that the digestive system cannot digest on its own [42]. Since the relationship among different species of bacteria is often competitive, this increase in energy-extracting bacteria occurs at the expense of bacteria that regulate other functions, such as combating inflammation [27]. These changes in microbiota may then lead to even greater sleep impairment, further slanting eating behavior, and instigating a vicious cycle [43].

A second direct pathway is the vagus nerve, which connects the brain to the gut’s intrinsic nervous system, called the enteric system. If rats have their vagus nerve severed, they are not affected by microbiome-related inflammation caused by sleep deprivation [44]. This indicates that the gut microbiome and sleep communicate, in part, in almost real time by way of the vagus nerve.

A third indirect pathway involves allostatic distress (Glossary). Sleep disruption increases the activity of the sympathetic nervous system and the hypothalamic adrenal pathway, increasing heart rate, decreasing heart rate variability, and increasing stress-related chemicals including catecholamines and cortisol [45]. Arousal-related catecholamines, primarily norepinephrine, and overactivation of the sympathetic nervous system can stimulate the growth of pathogenic bacteria such as *Escherichia coli* [46]. Aberrant sympathovagal drive, paired with catecholamines and glucocorticoids, may then change the microbiota habitat by increasing gut motility [47] and relevant iron availability [48].

### Therapeutic implications

With the multitude of pathways on offer, if an unhealthy microbiome impairs sleep, it follows that improving microbiome health may represent a novel therapeutic tool for improving sleep. While no causal interventions yet exist in humans, a recent study in mice offers early clues. Mice received a 4-week treatment of *Lactobacillus fermentum* PS150, a “psychobiotic” bacterium strain previously shown to reduce stress in rats [49]. At the end of the 4-week supplementation with *L*. *fermentum*, the mice were placed into the standard anxiogenic challenge of a new environment that reliably triggers sleep disruption [50]. The control mice displayed the typical reduction in NREM sleep caused by the anxiogenic challenge. By contrast, the mice who received the microbiome supplementation showed sleep resilience, suffering no such sleep impairment. While not a direct demonstration, it nevertheless hints at a functional pathway wherein improving gut microbiota may improve sleep. If correct, it may usher in a new concept of “physiobiotics,” here facilitating the physiological process of sleep (i.e., somnobiotics), beyond the psychobiotic field.

## Therapeutic enhancement of human sleep

**Fig 2:**
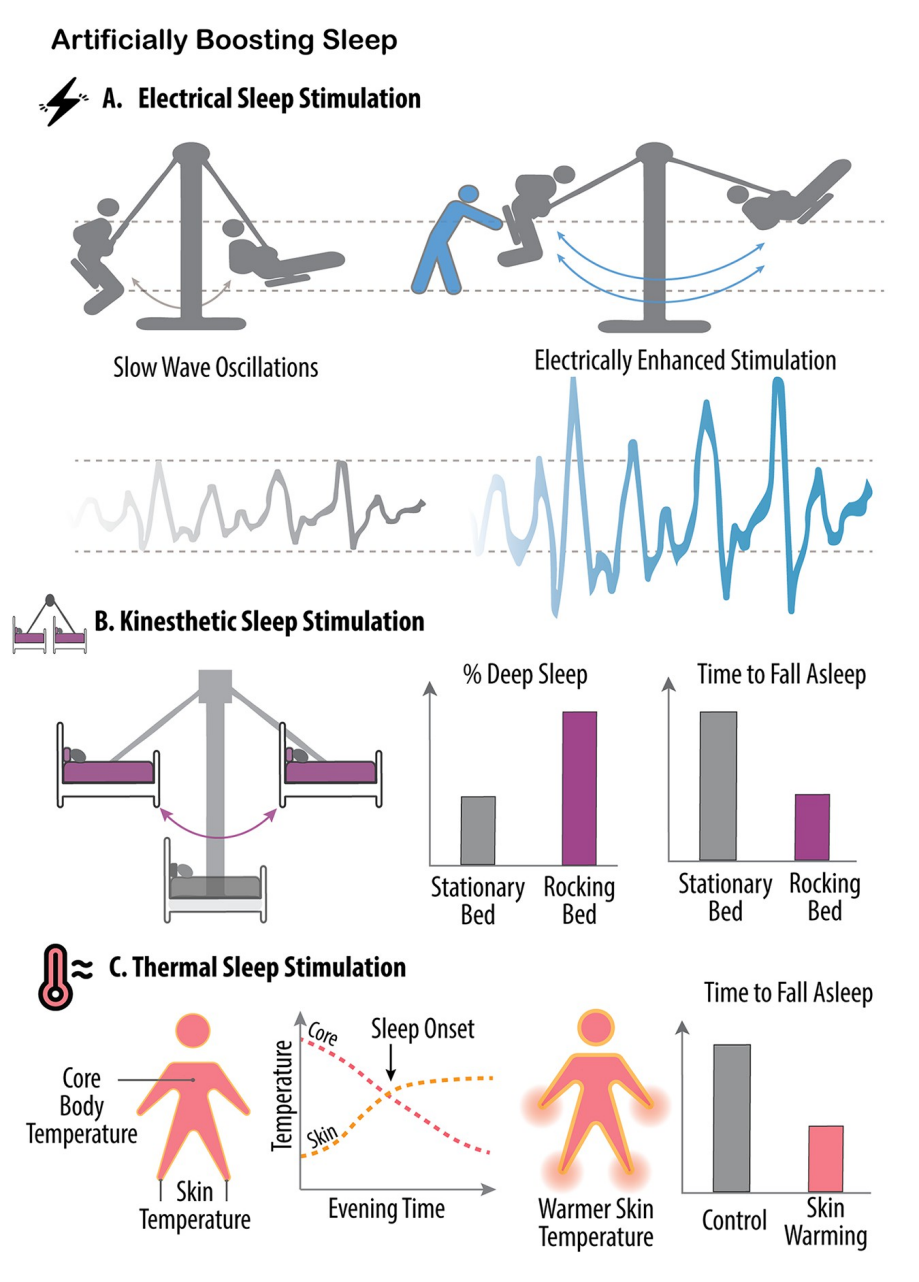
Artificially boosting sleep. Several different noninvasive methods have been developed for artificially augmenting human sleep. **(A)** Electrical brain stimulation, including when it is time-locked to the upcoming peaks of individual deep NREM sleep brain waves, can enhance the power of those slow waves in a mechanism similar to the external assistance, or pushing, of a swing. (**B**) A similar outcome can also be achieved by slowly rocking a bed at frequencies close to the slowest oscillations of deep NREM sleep (purple, approximately 0.25 Hz), leading to an increase in the amount of deep sleep, and helping with a faster sleep onset, relative to a stationary bed (gray). (**C**) Thermal stimulation of specific regions of the body represents another method for artificially improving human sleep. Normally, the mechanism instigating human sleep (sleep onset) involves an increase in skin peripheral temperature of vascular regions such as the hands and feet (yellow dashed line). As the blood rises to the surface away from the inner body, core body temperature decreases, and the coincidence of these 2 changes provides a thermal signal triggering sleep onset (red dashed line, left-side panel). Thereafter, further decreasing core body temperature is associated with increasing amounts of deep NREM sleep. By artificially accelerating these transitions, mostly by experimentally warming the hands and feet, core body temperature decreases more rapidly, therefore reducing the time it takes those individuals to fall asleep (right-side panel), with further such thermal intervention subsequently increasing the amount of deep NREM sleep and reducing the amount of nighttime awakenings (i.e., increasing sleep stability and the consolidated nature of sleep).

Throughout most industrialized nations, almost 1 out of every 3 individuals sleeps less than the recommended 7 to 9 hours of sleep per night [51,52]. Current pharmacological sleep aids have limitations and adverse effects [53] and the number of qualified individuals available to provide the behavioral alternative treatment of cognitive behavioral therapy for insomnia (Glossary) is limited, relative to the demand [54]. Thus, a need exists for new approaches that are cost-effective, low friction (i.e., interventions requiring minimal user effort or resources), have high compliance, and are scalable at a societal level. Emerging research developments, including electrical and acoustic brain stimulation, kinesthetic methods, and thermal manipulations, are beginning to show promise (Fig 2).

### Electrical sleep stimulation

Slow brain waves are the hallmark of deep NREM sleep and quickly became a natural candidate for approaches using noninvasive electrical brain stimulation. Early findings demonstrated that applying 1 Hz transcranial direct current stimulation during NREM sleep over the frontal lobe (a main epicenter of NREM SWA) can boost naturally occurring slow waves by up to 60% in healthy adults [55]. The sleep enhancement was meaningful, with individuals consolidating memories during this enhanced sleep in a superior manner, therefore forgetting less the next day [55]. A number of replication studies have since been published, though with some exceptions [56]. There has, however, been an unexpected change in sleep in the story of slow brainwave stimulation. In numerous electrical stimulation studies, the boost in SWA came with a secondary enhancement of sleep spindle activity, as was observed with kinesthetic rocking. This would indicate that, independent of the stimulation method (movement or electricity), when SWA is boosted, increases in faster-frequency bursts of sleep spindles also follow.

Although SWA has classically been linked to the enhancement of fact-based memory (i.e., textbook-like memory), so too have sleep spindles [57]. New closed-loop monitoring methods of electrical brain stimulation (i.e., a control system wherein the deviation signal of sleep is measured in real time and used to control the action of ongoing brain stimulation to fine-tune and perfect) have selectively targeted sleep-spindle frequencies [58]. Artificial sleep-spindle enhancements also led to superior next-day memory recall of previously learned facts [59], although interestingly, they did not improve motor-skill motor memories, which are also known to be improved by naturally occurring sleep spindles [58].

One of the most reliable and striking physiological changes as we age is a pernicious erosion of sleep, with a disproportionately large decline in deep NREM sleep [60]. This change is further exacerbated in those with dementia [61]. Considering the cognitive hallmark of aging linked to memory failure, and the fact that deep NREM sleep aids overnight memory consolidation, the direct health and disease applicability of electrical brain stimulation in older individuals and those with dementia has become a target. To date, electrical brain stimulation has, with some degree of consistency, improved the quantity and quality of deep NREM SWA in older adults, and those with dementia [62–64]. While large-scale randomized controlled trials are still required, transcranial stimulation is seen as promising since it is inexpensive and somewhat pragmatic and, therefore, scalable.

At least 2 applications have emerged. First is the use of electrical brain stimulation for facilitating healthy aging and potentially reducing the cognitive burden of dementia and/or enhancing glymphatic brain clearance of amyloid and tau proteins (see the section on “To sleep, perchance to cleans the brain”). Second, and lofty in speculation, is the question of whether such technology could offer a future in which we shift from a model of late-life treatment of aging and age-related disorders to a model of midlife prevention. It is during the fourth decade of human life that the decline in deep NREM sleep begins [65]. Starting a regimen of sleep augmentation at this time (e.g., as we commonly do with calcium supplementation to prevent osteoporosis) could help bend the arrow of age-related ill-health and dementia risk down on itself by maintaining life-long quality sleep.

### Acoustic sleep stimulation

Electrical brain stimulation still requires some degree of proactive motivation from an individual (applying the device each night, charging it, etc.). However, an alternative, low-to-no friction method for sleep enhancement is acoustic stimulation. When sounds are played without respect to ongoing slow waves, SWA is increased but memory retention is not improved [66]. A more sophisticated auditory stimulation approach has since emerged. Slow waves are detected in real time, with specialized algorithms predicting the timing peak of the next slow wave. At this time, a short sound is delivered to arrive at the peak of the next slow wave. This timed acoustic stimulation approach enhanced the expression of slow waves for some seconds after, and upon awakening, participants’ memory was 2-fold better compared with the unstimulated sleep nights [67]. A recent meta-analysis [68] has confirmed reliable and moderate effect sizes of acoustic sleep stimulation and the associated memory benefits.

### Kinesthetic sleep stimulation

In the human historical record, there is ample reference to rocking a small infant to invite sleep with alacrity. Several recent reports, in humans and nonhuman species, provide physiological data that support this long-known parental wisdom of kinesthetic sleep stimulation [69]. When healthy adults sleep on a bed suspended from the ceiling during a nap period, and the bed is then rocked laterally at an even, slow frequency of 0.25 Hz, sleep is enhanced [69]. Seeking to mimic the frequency of the very slowest NREM sleep slow waves, this 0.25 Hz stimulation had participants falling asleep significantly faster, spending less time in the shallowest stage of NREM sleep, entering a deeper stage of NREM sleep sooner, and obtaining more of that deeper sleep, relative to when they slept without the rocking motion. Deconstructing the sleeping brainwaves, the rocking method boosted the amount of ultraslow NREM sleep waves (0.5 to 1 Hz) and increased another physiological bursting oscillation often paired with these slow waves, called sleep spindles (10 to 15 Hz).

Recently, these findings were replicated across a whole night’s sleep [70], with the study further showing that these rocking-induced benefits also had functional effects. Participants performed almost 10% better on a memory test after sleeping on the rocking bed compared to when sleeping on the stationary bed (not dissimilar to a full grade increase on an exam). Similarly, mice that were rocked gently by having their cage placed on a moving platform fell asleep faster, and spent more time in NREM sleep, although without changes in brainwaves. Elegantly, when the same experiment was performed on mice lacking sensitivity to linear movement, they did not experience any changes in their sleep patterns, confirming that it is the kinesthetic movement that augmented the sleep benefit [71]. By employing a vibrating pad, set at a specific frequency, even fruit flies can be lured into slumber [72]. Interestingly, with each repetition of the rocking procedure, the flies fell asleep more rapidly. However, this enhancement occurred only when the frequency remained unchanged; any slight alteration prompted the flies to reacquaint themselves with the new rhythm.

These latter findings suggest that the process of getting used to sensory stimulation helps in reducing arousal levels and, thus, promoting sleep [72]. More generally, the idea that a slow rocking kinesthetic improves sleep has already spurred the development of at least 1 commercial appliance at the time of writing this article. The device—essentially a set of 4 sturdy motor-driven movement pads—is placed under the feet of the bed. The pads instigate a rocking motion at the aforementioned slow frequency with the hope of sleep improvement (Enseven LLC, Arizona, United States of America).

### Thermal sleep stimulation

If you isolate an individual from time and context cues, they will unwittingly report the greatest natural urge to sleep precisely when their core body temperature begins to plummet [73]. Temperature, therefore, offers 1 novel and newly harnessed pathway for enhancing human sleep [74,75]. The main evidence for this comes from pioneering work carried out by a team of sleep scientists led by Eus van Someren [76]. The team ingeniously developed a thermal bodysuit filled with tubes, much like veins, capable of selectively perfusing water of different temperatures to any specific part of the body. To artificially accelerate a drop in core body temperature, the scientists first focused on increasing the temperature of the peripheral extremities (hands, feet, arms, legs). When these peripheral areas are warmed, blood rises to the skin’s surface. As a result, warm blood from the inner core of the body is encouraged outward, allowing the rapid expulsion of core body heat, dropping central body temperature, and thereby inducing sleep. By controlling the temperature of the perfused water, they effectively accelerated the natural temperature drop that facilitates sleep (i.e., peripheral body warming to cause core body cooling; Fig 2). As a result, they had participants falling asleep approximately 25% faster than was normal for them. As they continued to mimic the body’s natural thermal sleep change further into the night, more sleep benefits unfolded. By continuing to cool the body into the first half of the night using the same suit, the scientists reduced the amount of time awake, thus increasing the amount of time spent in stable sleep, and the electrical quality of deep NREM sleep also increased [76].

Elderly individuals are one population that struggles with sleep and thermoregulation. Van Someren and colleagues have since targeted these older adult populations [77]. Before the body-cooling therapy, older adults in the study had more than a 50% probability of waking up in the last half of the night. After applying the thermal cooling manipulation throughout the night, the number decreased to less than 5% likelihood, and deep NREM sleep also increased.

Of course, thermal suits are not scalable owing to high cost and low compliance. However, baths and showers are a simpler, cheaper, and accessible alternative. Upon exiting the warm bath or shower, heat is again expelled faster and more efficiently from the body than without either of these thermal manipulations, leading to a drop in core body temperature [78]. A collection of studies utilizing warm baths or showers before bed [78–81] have, on average, resulted in individuals falling asleep between 10% and 30% faster, having fewer awakenings at night, and increasing the amount of NREM sleep by 50 additional minutes, relative to nights without prior hot bath or shower interventions. There may be a cost though. Some studies have reported a co-occurring decrease in REM sleep following hot baths or showers, either due to the NREM increase or to a change in body temperature shifting away from that which is optimal for REM sleep. Notably, manipulating REM sleep using temperature has been achieved by changing the ambient temperature in the room of the sleeper, rather than skin temperature. Absent sheet bedding, when the ambient temperature is close to thermal neutrality for endotherms (which, for humans, is between 29°C and 31°C, or 84°F and 88°F), REM sleep is maximal [82,83]. In rodents, when the ambient temperature is increased from 22°C to 29°C, moving more toward the thermal neutral zone, REM sleep more than doubles [84,85]. Nevertheless, the effect follows an inverted U-shape function, with REM sleep decreasing back down if the temperature is increased to 36°C [84,85]. Consumer technology groups have taken note. Smart home thermostats for ambient room temperature, and thermal-modulating mattresses controlling the temperature below the covers, all offer scalable approaches to altering human body temperature during sleep, although no formal peer-reviewed articles have been published to date.

### Novel pharmacological sleep aid

Another development for enhancing REM sleep has emerged from the pharmacological arena. Over the past decade, drugs targeting receptors for orexin (also known as hypocretin) have emerged. Orexin is a neuropeptide that stimulates wakefulness and food intake [86,87]. These drugs, known as dual orexin receptor antagonists (DORAs), block both orexin receptors (OX1 and OX2), thereby inhibiting wakefulness and promoting sleep. Unlike previously developed hypnotic drugs, such as benzodiazepines and Z-drugs, which predominantly augment NREM sleep in a sedative-hypnotic manner, DORAs promote a different sleep signature. The 3 dominant DORAs (suveraxant, lemborexant, and daridorexant) not only enhance sleep by reducing sleep onset latency, wakefulness after sleep onset, and total sleep time [88], but these medications also reduce the time to the first appearance of REM sleep and increase the total amount of time spent in REM (suverxant [89], lemborexant [90], Deoraxant: N/A). Surprisingly, only 1 study (looking at suverxant) has published electroencephalogram (EEG) spectral data that offers insight into the effect of the drugs on sleep oscillations [91]. No significant changes to electrical EEG activity in REM or NREM sleep were observed at any of the wide-ranging doses of the drug used (even after 28 days of use). While preliminary, such data suggest that the DORAs consolidate and lengthen sleep without altering its fundamental oscillatory characteristics of cortical activity. Notably, when administered to older adults with suspected Alzheimer’s disease, suverxant increased sleep duration by 73 minutes per night (28 minutes more than placebo) [92]. In addition, recent findings in a small group of unimpaired middle-aged adults indicated that suverxant use decreased amyloid-β levels overnight by 10% to 20% in the cerebrospinal fluid (CSF) [93], the consequences of which we discuss in the next section.

## To sleep, perchance, to cleanse the brain?

The body’s cleansing system, or lymphatic system, was first described in the 17th century by Olaus Rudbeck and Thomas Bartholin [94], yet the existence of any such cleansing system within the brain was not discovered until 1984, when Patricia Grady and Marshall L Rennals replaced the CSF of anesthetized cats and dogs with a tracer solution that could be tracked in brain slices under the microscope [95]. Still, it was only in 2013 that a team of researchers led by Maiken Nedergaard published a landmark set of discoveries that associated this cleansing system with sleep and postulated that it may explain why animals (or metazoans) with nervous systems require sleep.

The glymphatic system of the brain is made up of a matrix of glial cells that are nonneuronal in nature and utilize a set of water channels called aquaporins on their end feet [96]. Glial cells combine to form a space around the brain’s vasculature, called the perivascular space, in which CSF flows [97–100]. The glymphatic system services the removal of metabolic detritus, solutes, and toxins from the brain, specifically from the interstitial space between neural cells [98].

### Sleep and the glymphatic system

Nedergaard and colleagues’ discovery, together with the contributions of many others [96,101], has established that the pulsing, cleansing glymphatic mechanism is not always switched on in high-flow volume across the 24-hour period. Instead, it is during sleep, and particularly during NREM sleep, that the glymphatic system shifts into full tempo. A seminal study in mice utilized CSF tracers to measure the CSF pulsing flux throughout the brain. When the mice were awake, CSF flow was minimal; however, when the mice entered NREM sleep, CSF flow increased considerably [97]. Strikingly, the extracellular space between the brain’s cells and structures (interstitial space) increased by 60%. As a result, there was markedly greater CSF flow coursing through the interstitial space, enhancing the exchange of waste products between the CSF and brain cells. Two notable waste products removed are amyloid-β and tau proteins, the excess accumulation of which is the hallmark of Alzheimer’s disease, and which we will return to in the section on “Disease implications” [102].

In humans, various studies have demonstrated that sleep has a causal role in removing waste products from the brain. Depriving individuals of sleep for an entire night, or even just selectively reducing the amount of deep NREM sleep (while holding a constant total sleep time), results in a next-day increase in amyloid-β and tau. This has been measured by markers in the circulating bloodstream [103], within the CSF (assessed using lumbar puncture) [104], and directly in the brain using amyloid-β- and tau-sensitive PET scans [105].

### Sleep-dependent mechanism

Why is the sleep state essential for glymphatic clearance? First, the high levels of brain noradrenaline that dominate during arousal drop during sleep. Within the brain, one structural consequence is that the interstitial space expands [97], allowing for better-flowing conditions. Second, cardiorespiratory oscillations change markedly during NREM sleep. Both cardiac and respiration cycles slow down, respiration becomes deeper, and the temporal coupling between the two increases [106]. These pulses drive the mechanical contraction and dilation of the blood vessels, which, in turn, results in a corresponding and respective expansion and shrinkage of the space surrounding the vessels in which CSF resides [100]. Indeed, these cleansing fluctuations are 2 to 5 times larger in NREM sleep relative to the waking state [107]. Third, recent studies show that neural activity itself might influence CSF flow locally [108]. When neural activity decreases, the demand for fresh oxygenated blood decreases as well, which translates to narrower surrounding blood vessels and wider perivascular spaces that fill with CSF [108]. During NREM sleep, brain activity shows synchronous rhythmic SWA spanning vast brain areas, as opposed to the faster and desynchronized brain activity observed during wakefulness. The newly discovered involvement of brain activity affecting CSF flow could explain how spatially coordinated and rhythmic neural activity during NREM sleep, as opposed to the erratic and spatially diverse metabolic demands during wakefulness, supports efficient cleansing by synchronously widening the vascular space across larger brain territories.

The majority of mechanistic data illustrating the sleep-dependent operation of the glymphatic system has been in mouse models. However, a recent seminal study in humans employing a novel functional MRI (fMRI) marker to measure the strength of the CSF flow signal has provided the first hints of the same mechanistic system at work. As participants went into NREM sleep inside the MRI scanner, a significant increase in CSF flow was observed at the fourth ventricle, a large CSF cavern deep in the brain. Interestingly, this surge in CSF flow was preceded by a coupled increase in whole-brain oxygenated cerebral blood flow, which was, in turn, preceded by the electrical SWA that is prevalent in NREM sleep [109]. Thus, a physiomechanical rhythm creates a corresponding pulse and flow of CSF fluid, thereby representing a sleep-dependent pathway that supports the glymphatic sanitary service.

### Disease implications

Impaired glymphatic clearance has been described and/or proposed in a collection of neurological disorders, including Alzheimer’s disease, traumatic brain injury, and Parkinson’s disease, as well as in psychiatric disorders [110]. Of note, every one of these conditions has well-established impairments in sleep. Of these, the most studied is the relationship between impaired sleep, Alzheimer’s disease, and the glymphatic system [89].

Hour-to-hour fluctuations in amyloid-β levels across the 24-hour period correlate strongly with the sleep–wake cycle in both mice and humans, rising during the wake phase when sleep is absent, and declining during the sleep phase when sleep occurs. However, mouse models of Alzheimer’s disease, in which sleep is impaired, do not show such diurnal fluctuations [111], suggesting that appropriate waste clearance is not taking place due to deficient sleep. Relatedly, mice whose sleep is pharmacologically suppressed for 9 hours experience a 2-fold increase in tau levels [112] and a 17% increase in amyloid-β levels within the brain [111]. In humans, the lower average duration of sleep across the life span, together with the disorders of sleep apnea and insomnia, are all associated with increased amyloid-β levels in later life and/or with a higher risk of developing early cognitive decline and Alzheimer’s disease. Moreover, there is a progressive linear impairment of fMRI-measured CSF flow—a proxy for aspects of glymphatic activity—in later life, with the severity of impairment increasing with the transition in older adults from health, to those showing signs of mild cognitive impairment, and, finally, to those with Alzheimer’s disease [113]. Showing bidirectionality, treating sleep apnea in midlife delays the onset of cognitive decline by over a decade [60,114].

While these data offer an explanatory mechanism for the well-known link between insufficient sleep and Alzheimer’s disease, they also raise the question of sleep as therapy. If the decline in deep NREM sleep, which begins as early as the fourth decade of human life [65], can be prevented, one could conceivably be able to decrease Alzheimer’s disease risk.

## Sleep and emotional health

Any parent knows that poor sleep in a child the night before leads to poor emotional reactivity the following day. The same, it turns out, holds true for adults. Insufficient sleep quantity, quality, and select NREM and REM sleep abnormalities are associated with emotional dysregulation, anxiety, aggression, and worse mood (effect-size range g = 0.39 to 0.94) [115–118]. Recent neuroimaging studies have further revealed a unique neural mechanism accounting for these alterations in mental health caused by a lack of sleep [119–121]. Most intriguing, the sleep manipulations used to produce these affective changes in healthy adults mimic those expressed in specific psychiatric and neurodevelopmental disorders, including major and bipolar depression, anxiety, schizophrenia, autism spectrum disorder (ASD), and attention deficit hyperactivity disorder (ADHD) [122–126]. Indeed, no major psychiatric disorder has been studied to date in which sleep is normal [124].

### Sleep loss and emotional health

Three key domains of affective brain function become compromised when sleep becomes short or of poor quality: mood and emotional baseline; (mis)perception of other people’s emotions; and an individual’s outward emotional expressivity to other people.

Concerning the basic tenor of an individual’s emotional baseline, complete or partial sleep restriction worsens mood states and increases emotional reactivity. Consequently, negative feelings of anxiety, agitation, hostility, anger, and restlessness [116,117,127,128] and, to a lesser degree, impulsivity [129–131], are increased. However, the adverse effect of a lack of sleep on blunting positive emotions is even greater than that of amplifying negative mood. Almost all dimensions of positive mental health diminish with insufficient sleep, including feelings of happiness, excitement, energy, motivation, and the general ability to gain pleasure from normally pleasurable experiences (anhedonia) [115,117,132,133]. Sufficient research enabled 2 very recent meta-analyses to be performed that quantify how sleep compromises mental health. A large effect size was found for the blunting of positive affect by sleep loss (g = −0.94, *n* = 25 studies), while increases in negative mood and increases in anxiety were also robust, although less pronounced (g = 0.45, *n* = 55 studies; and g = 0.39, *n* = 34 studies, respectively [115,116]). Interestingly, recent data indicate an important role of sleep regularity in protecting better mood and emotional health [134]. For example, increased variability of sleep duration (as measured across a week) predicts lower satisfaction with life, greater depressive symptoms, and increased anxiety [135]. Similarly, variability in sleep timing from day to day precedes poor mood, and worsening mood the following week, and does so independently of age, sex, level of physical activity, and sleep duration [136]. These findings collectively support the realization that, in addition to sleep duration and quality, the consistency of sleep can also be linked to numerous mental health outcomes.

Beyond dulling pleasure while increasing states of negativity, sleep loss also impacts the intensity with which these emotions are experienced [137]. When facing a modest cognitive challenge (such as counting backward in steps of 2), sleep-deprived participants will rate it as more stressful than those who had a night of sleep [132]. This suggests that sleep loss changes the internal cutoff or emotional threshold the brain uses to determine our transition into emotional distress. As a result, sleep restriction, poor sleep quality, and irregular sleep have all been linked to heightened subjective stress [137–139], an association that is only exaggerated in children with ADHD or ASD [140]. Notably, sensitivity to stress is known as the “lowest common denominator” that promotes vulnerability, or exacerbates symptoms of almost all mental illnesses, the majority of which include sleep loss or insomnia as part of their diagnostic criteria [141–143].

**Fig 3:**
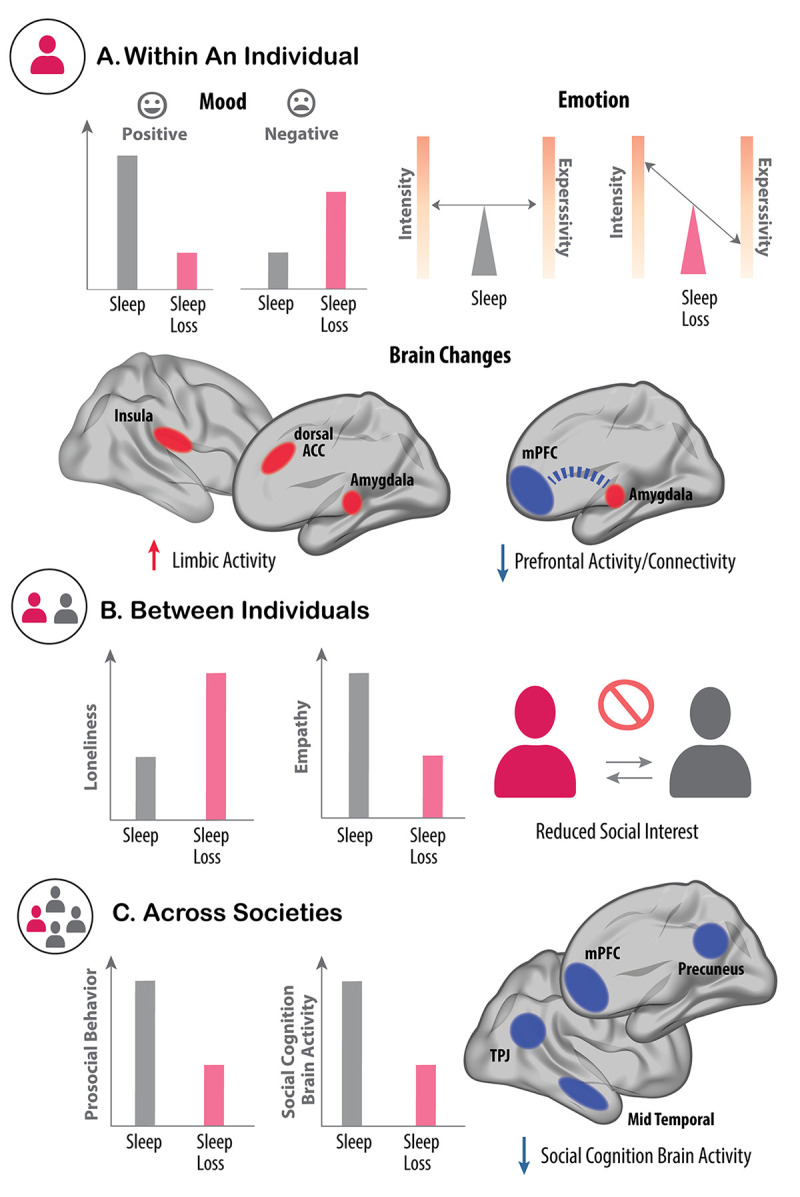
Social and emotional consequences of sleep loss. **(A)** Within an individual, sleep loss (pink) triggers a sharp reduction in positive mood and, to a lesser extent, an increase in negative mood (left-side panel). The emotional intensity felt by sleep-deprived individuals is also amplified by lack of sleep. However, there is a paradoxical decrease in the outward emotional expressivity triggered by sleep deprivation (right-side panel). These affective changes are further reflected in the brain. Here, sleep loss increases activity in the limbic network involved in emotional processing (red, left-side brain) yet reduces activity in the mPFC (blue, right-side brain). In addition, functional connectivity between the mPFC and amygdala is also reduced by sleep loss (dashed blue line), which is a communication pathway that normally regulates emotion. (**B**) Interindividual affective processes and behaviors are also altered by sleep loss. For example, sleep loss increases feelings of loneliness within the sleep-deprived individual and lowers feelings of empathy towards others (left panel). This asocial phenotype within an individual is further reflected in the reduced desire to interact with other, rested individuals. This effect is bidirectional. Rested individuals, unknowing of the sleep-deprived state of their conspecific, nevertheless show a similar reduction in the desire to interact with underslept others (right panel). (**C**) Across larger societal scales, insufficient sleep impairs prosocial behavior observed in large groups of individuals. For example, underslept groups express a reduced overall trend of helping behaviors and reduced motivation of typical societal civic duties, such as volunteering or voting (left panel). One underlying mechanism accounting for these collective asocial consequences is impaired activity in the social cognition brain network of underslept individuals (right panel), which is relevant as this network normally supports the ability to understand the state of others (i.e., theory of mind), and also promotes prosocial helping and cooperation. ACC, anterior cingulate cortex; mPFC, medial prefrontal cortex; TPJ, temporal parietal junction.

The underlying mechanisms explaining these changes in our innate emotional balance have been linked to aberrative physiological changes to the brain and body. Within the brain, sleep loss increases limbic reactivity and decreases functional connectivity (Glossary) between the medial prefrontal cortex and limbic structures, thereby diminishing emotion regulation capabilities (Fig 3A) [133,144–147]. Notably, the neural circuit connecting the amygdala to the anterior cingulate cortex has recently been shown to protect against mood disruption triggered by one night of sleep deprivation in both healthy individuals and those with depression [145]. Such findings indicate that changes to amygdala connectivity following a lack of sleep have a significant role in shaping both emotion and mood regulation without sleep. These changes in connectivity can be viewed more generally as confirmatory to the synaptic homeostasis hypothesis [148], suggesting that one function of sleep may be to rebalance or downscale synaptic strength that is potentiated during the day.

Potentially related to the aforementioned changes in limbic brain connectivity, or contributing to them, are increases in autonomic pupillary reactivity, increased skin conductance, up-regulated cortisol release, and increased blood pressure following lack of sleep [149–154]. Such a collection of changes suggests an explanatory biological framework of skewed brain–body sympathetic drive in response to emotionally inciting events, although, paradoxically, when quiescent, a new study has shown an opposite pendulum swing to excess parasympathetic drive under sleep-loss conditions [155]. Similarly, habitual short sleep (<7 hours) has recently been linked with reduced amygdala reactivity compared to normal sleep duration (7 to 9 hours), potentially indicating long-term desensitization of limbic reactivity following chronic insufficient sleep [156].

In addition to internal emotional feelings, contemporary work has established that emotional perception becomes skewed when sleep is insufficient. As a result, individuals can perceive a distorted view of incoming emotional signals from others and from the world.

Following reductions in either sleep quantity or quality, participants pay greater attention to and react faster to negative emotional stimuli (relative to neutral or positive stimuli) [129,157–159]. More than a change in the rose tint of an individual’s emotional perception is the recent discovery of emotional misperception. Here, sleep-deprived individuals fail to discriminate accurately between the different gradings of emotional facial expressions [160,161], with sleep loss biasing individuals to perceive greater threat signals relative to safety [162]; i.e., sleep-deprived individuals will more commonly mistake friend for foe [163], consistent with the proposal that the underslept brain loses the appropriate “tuning curve” of accurate emotional stimulus discrimination [45]. Nevertheless, when using short clips of individuals enacting varied emotional expressions, rather than still images, sleep loss does not significantly impair emotion recognition [164]. One explanation for this is that richer, more dynamic stimuli might sufficiently heighten attentional focus, motivation, or levels of arousal to compensate for the otherwise observed discrimination impairments triggered by lack of sleep.

The third domain of emotional function altered by a lack of sleep is one uncovered only recently, perhaps in part because it was so paradoxical. Contrary to the prediction one would make based on the internal sensation of amplified negative emotions, combined with the physiological sympathetic and limbic reactivity, the outward emotional expressiveness of underslept individuals is stunted (Fig 3A). This has been demonstrated across the emotive level of vocal expressiveness and facial expressivity [165–169]; e.g., sleep loss reduces pitch variations when individuals speak, making their voice sound more monotonic or “flattened” [165,166]. Therefore, sleep-deprived individuals experience increased emotional sensitivity themselves yet suffer a paradoxical outward reduction in expressivity (of that amplified emotional state).

There are many implications for these discoveries. One of the most powerful ways that human beings communicate nonverbally is through emotional behavioral expressions (e.g., voice, face, movement) [170]. Consider a sleep-deprived patient in a hospital not being fully communicative of their pain state and, thus, not being given appropriate pain treatment by medical staff (particularly relevant as sleep-deprived individuals feel noxious stimuli as more painful relative to when they are well rested [171–173]). Indeed, this very absence of signaled outward expression may explain why sleep-deprived participants are routinely viewed as less desirable to interact with, propagating the impact of sleep loss into the social domain (as we discuss below).

### Benefits of sleep for emotional health

Sleep loss and sleep restriction lead to clear detrimental effects on our emotional well-being. The latest work has inverted the question: What is it about sleep, when we do get it, that beneficially improves mental health? Initial findings highlighted the role of REM sleep in the support of emotional processes [174–177] and in providing a form of overnight therapy, dissipating the subjective intensity of emotion when individuals are reexposed to an emotionally challenging event from the previous day [178,179].

However, the most recent findings have offered a revision of this REM sleep focus, establishing a role of NREM SWA in offering complementary effects on the mood state of anxiety, more than moment-to-moment emotional reactivity. More specifically, the amount of time spent in deep NREM sleep, as well as the electrical brainwave quality of that deep sleep indexed in SWA, service an overnight amelioration of anxiety in healthy adults, returning it to baseline levels. The greater the amount and quality of NREM SWA, the less anxious the individual felt the next day. When sleep was absent, however, anxiety progressively increased across the night and into the next day [128]. Interestingly, the underlying neural mechanism associated with this deep-sleep anxiolytic effect was somewhat similar to the effects of REM sleep. Both the amount and the quality of SWA predicted the extent of medial prefrontal cortex reengagement the next day, a region essential for the down-regulation of anxiety, and which is impaired in those with anxiety disorders (who also have co-occurring deficiencies in NREM sleep) [180–182]. Sleep, and the unique biological states of REM and deep NREM, may therefore explain the prophetic wisdom of American entrepreneur, Joseph E Cossman, who once declared, “The best bridge between despair and hope is a good night’s sleep.”

## Sleep-dependent prosocial control?

Humans are a social species, psychologically and biologically requiring social connectedness. Collectively, as a species, survival necessitates such social, interindividual cooperation [183]. Indeed, without prosocial cooperation and helping, the advent of modern societies would not have occurred.

Sleep is a fundamental prosocial glue that binds human beings and entire societies together. The impact of sleep, and a lack thereof, has now been elicited from the level of a single individual’s social proclivity (e.g., social approach, social withdrawal, and loneliness) through to the prosocial interactions between humans (including the complicated processes of empathetic understanding and cooperative helping), and all the way up to the en masse coordination of societal behaviors (Fig 3).

### Sleep loss and the (a)social individual

Within an individual, a lack of sleep leads to feelings of social disconnection and loneliness. Insufficient sleep, including that caused by insomnia, poor sleep quality, difficulty falling asleep, and greater daytime sleepiness, are all associated with greater loneliness and a reduced desire to interact with others [184–187]. Moreover, sleep loss changes the way individuals evaluate their own social experiences, reducing a sense of connectedness and related positive affect and reducing the desire to interact further [188]. In longitudinal studies, initial poor sleep quality (including sleep fragmentation) and lower sleep satisfaction are predictive of higher levels of loneliness 2 to 7 years later [189,190], while preexisting loneliness is predictive of worsened subjective sleep quality, highlighting the bidirectional link between sleep and social isolation [186].

By contrast, superior sleep quality, including an ability to fall asleep more quickly with fewer nighttime awakenings, is associated with a higher likelihood of daytime active socializing [191]. This relationship is especially true regarding prior NREM slow wave sleep (SWS), with greater amounts and quality of SWS resulting in increased amounts of real-world social interactions the following day [192]. Offering bidirectional evidence once again, the social isolation of mice triggered a significant decrease in sleep amount, most notably reductions in the electrical quality of deep NREM sleep [193,194]. Thus, insufficient sleep, specifically reduced amount and electrical quality of NREM, can lead to a behavioral phenotype of social withdrawal and loneliness, while loneliness and social isolation instigate impairments in sleep quantity and NREM quality—a self-perpetuating cycle. Yet, REM sleep also appears highly relevant. Recent work has established a causal role for REM sleep in the consolidation of social memory [195], such that REM-specific suppression of hippocampal neural circuits in sleeping mice lowered the typical preference for novel social interaction the next day [196]. Similar impairments in social novelty preference were also recently observed following sleep disruption in adolescent mice, an effect that was linked to impaired reward-related dopaminergic activity when meeting a new conspecific [197]. Together, such findings indicate that sleep disruption of numerous kinds and stages leads to a phenotype of social withdrawal and disengagement driven by sleep-dependent neural circuits that otherwise sustain adaptive prosocial behavior.

### Sleep and interpersonal social interaction

In addition to changes within an individual, interactions between individuals are also dependent on sleep (Fig 3B) [119,198,199]. Among romantic partners, poor sleep quality is associated with greater conflict the following day, higher levels of aggression, and lower marital satisfaction [200,201]. In children and teens, poor sleep quality predicts increased hyperactivity, more conduct problems, more disagreements with peers, more violent behavior, and a greater propensity for bullying [202–204]. Similar outcomes are observed in children with ASD, in whom short sleep duration and poor sleep quality are also related to difficulties in social interactions and fewer prosocial behaviors [205,206]. Notably, improving sleep in individuals with ASD can alleviate their symptoms, increase social communication skills, improve appropriate emotional reactivity, and decrease maladaptive and repetitive behaviors [207].

Further leading to the interindividual erosion of social bonds by a lack of sleep, underslept individuals are rated as less interesting or desirable to interact with by well-rested individuals, even when those well-rested individuals know nothing about the sleep status of the people they are rating [208,209]. Sleep-deprived individuals are further rated as lonelier, less attractive, less charismatic, more anxious, and more unhealthy-looking by independent judges who are similarly blind to the sleep status of those individuals they are rating [184,210]. This suggests that sleep deprivation curates a form of individuals who are socially repulsing (in the literal sense of the word) to the rest of society.

Our workplaces also suffer the deleterious impact of sleep loss on social functioning. A lack of sleep decreases the extent of helping behavior among colleagues in the workplace [211,212] and raises levels of overall hostility between employees. Morality suffers, too. Underslept employees show a significantly higher probability of unethical behaviors, such as blaming someone else for their own mistakes, or dishonestly taking credit for someone else’s work [213]. The social disconnection between individuals that ensues from a lack of sleep has also been identified within the hospital setting, to ill effect. Doctors who have insufficiently slept when working a night shift are significantly less empathetic to their patients’ pain and, as a result of this deficient empathy, prescribe fewer analgesic medicines to help alleviate patients’ pain, relative to doctors working a day shift [214].

A new development has added a peculiar feature to our understanding of sleep’s influence on interindividual dynamics. When a well-rested individual interacts with an underslept individual, the nonverbal signals of loneliness emitted by the sleep-deprived participant can be “transmitted” to the well-rested individual, making the well-rested person feel lonelier themselves [184]. Such virus-like propagation from sleep-deprived to well-rested conspecifics intimates that the ill effects of sleep loss can spread to nearby social circles and further aggravate loneliness, leading to a wider-reaching impact of insufficient sleep on social withdrawal.

### Sleep and society

Moving beyond interpersonal interactions, new developments point to an influence of sleep loss in altering the unique societal forces that shape human communities. Humans help each other—helping is a fundamental aspect of social humanity and one that is eroded by a lack of sleep [215]. For example, decreasing sleep simply by 1 hour diminishes helping acts of civic engagement, such as signing petitions and volunteering [216], and reduces the likelihood of voting across multiple different nations [216,217]. Insufficient sleep, be it total deprivation or simply modest night-to-night fluctuations in sleep quality, also leads individuals to withdraw their normal proclivity to help others [215] (Fig 3C). One study examined over 3 million charitable donations made in the USA in the past decade. The loss of 1 hour of sleep opportunity, using the manipulation of the change to Daylight Saving Time, substantially decreased altruistic helping across all states that undergo a clock transition [215]. This same dent in compassionate gift-giving was not seen in regions of the country that did not change their clocks and, thus, whose sleep was uncompromised.

How a lack of sleep produces this potent impact on human sociability appears to be driven, in part, by alterations in brain networks that compute and make complex social choices. The social cognition network, which involves regions of the medial prefrontal cortex, mid and superior temporal sulcus, temporal–parietal junction, and the precuneus [218–220], helps support social computation and, consequently, decisions on appropriate prosocial actions [221–223]. Two recent studies have shown that a lack of sleep impairs the activity and social responsivity of this network [184,215]. Furthermore, the magnitude of impairment predicted a greater withdrawal of choices to help others [215], suggesting a neural basis for asociality when sleep gets short. Such an effect of sleep on the higher-order complex social computations of the brain remains even when taking into account changes in negative mood and motivation. Moreover, sleep loss could stunt the altruistic helping nature of the individuals in a manner that discounted close social bonds, such that participants who had had insufficient sleep withdrew their help to others regardless of whether those in need were strangers or people they personally knew, such as close friends or family members. These results suggest that sleep loss can trigger a phenotype of asocial behavior with a broad and indiscriminate impact.

Parenthetically, data have indicated a steady decline in empathy behavior and civic participation in the USA over several recent decades [224,225] that is paralleled by declines in sleep quality and aspects of sleep quantity across the same time period [226,227]. Reductions in sleep quantity and quality in industrialized nations may thus be a previously unconsidered factor contributing to some asocial trends.

## What is in a dream?

Each night, individuals experience a state of altered consciousness known as dreaming. At times, they are notably disorientated, losing track of time, place, and person. They experience hallucinations, perceiving things that are not present, and show signs of being delusional, believing things that are clearly not possible. Added to this are large emotional pendulum swings, vacillating between extreme positive and intense negative emotions. Finally, upon awakening, they endure a degree of amnesia, forgetting large segments of the bizarre journey that has just happened, if not the entire experience. If this was not peculiar enough, almost all of this experience unfolds without any volitional control. This is the state of dreaming, and since the record of human species began, dreams have been a noted part of it [228]. However, only recently have sleep scientists begun to understand some fundamental aspects of dreaming, including how human brains dream and if other species show similar neural instantiation of the dream state; what, if any, function(s) dreams serve (above and beyond the state of sleep they come from), leading to the development of dream therapies to restore these benefits; how to “mind read” the dreams of others using fMRI; and if and how individuals volitionally control their dreams (known as lucid dreaming).

### How the brain dreams

**Fig 4:**
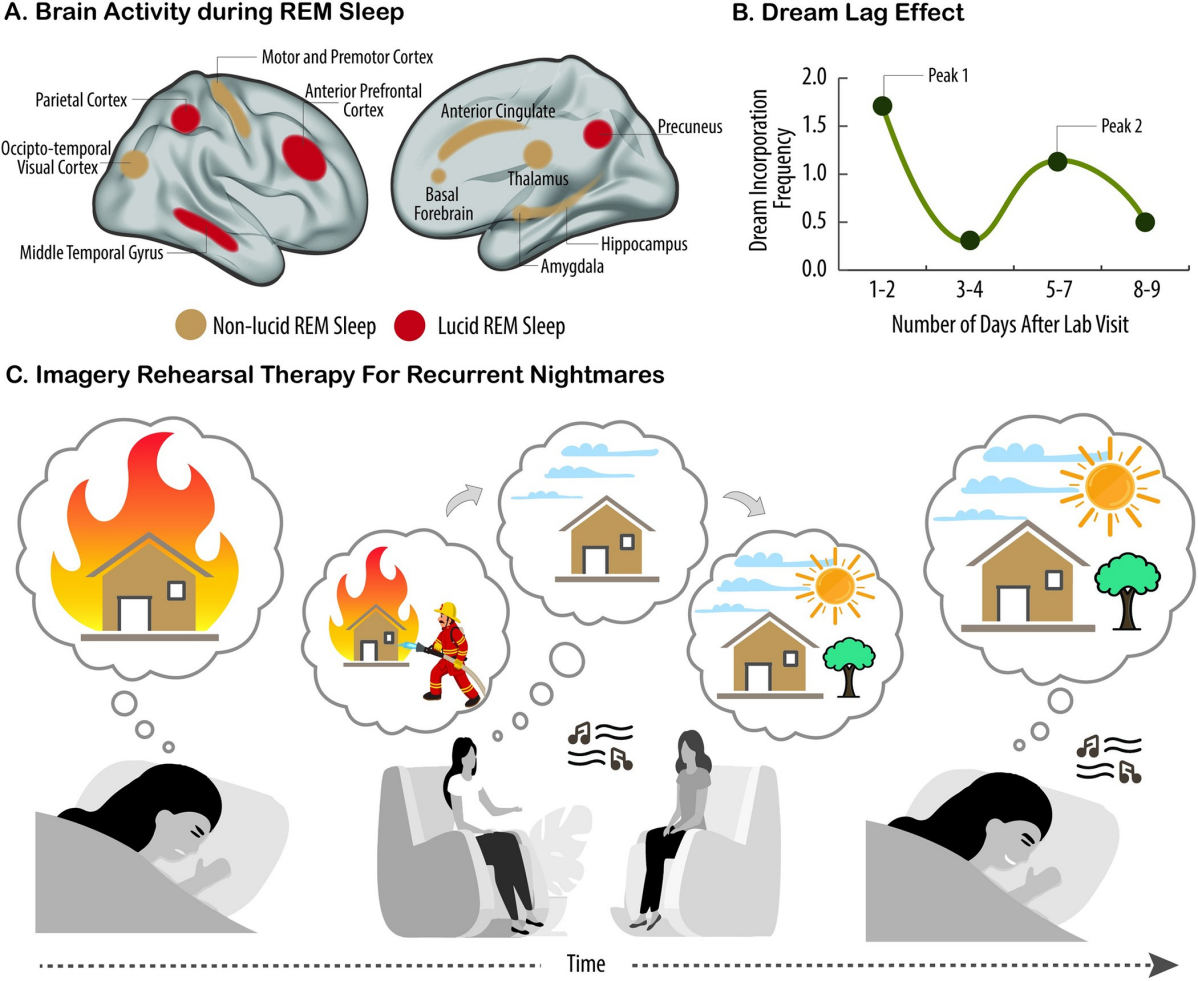
Dreaming and the brain. **(A)** Brain activation during REM sleep—one of the principal stages associated with vivid dreaming. Relative to brain activity when an individual is either awake or in non-REM sleep, there is increased activation of visual, sensorimotor, and affective pathways during REM sleep (golden clusters). Additional regions then come online and are activated when individuals experience lucid REM sleep (red clusters; relative to nonlucid REM sleep). These include regions of the anterior prefrontal cortex involved in volitional executive decisions and actions, and the precuneus, involved in self-referential processing. (**B**) Incorporation of recent waking events into dreams unfolds in a 2-peak reliable pattern over time. The first temporal peak of waking incorporations occurs on the first 2 nights and then fades. However, these same prior waking experiences reemerge as a second peak 5–7 days later. This temporal pattern of waking life incorporation is known as the dream lag effect. (**C**) IRT is a behavioral intervention method for treating and dissipating nightmares. IRT includes the waking rehearsal of alternatives to nightmare scenarios, developed between the patient and their therapist. These more neutral or positive alternatives to the nightmare scenario are rehearsed daily by the patient for up to 2 weeks. As a result, the nightmares become significantly less distressing. A recent study added an additional methodological step. During the daytime rehearsal of the nightmare alternative, an auditory tone (here, a piano chord) was played every 10 seconds in the background. Then, as the patient slept and went into REM sleep—the stage most commonly associated with nightmares—the same piano chord was played at a level that did not wake the patient up. The goal was to reactivate the memory of the alternative scenario as the sleeping brain is processing. As a result, patients experienced an even larger decrease in the distressing nature of the nightmare, relative to standard IRT. IRT, imagery rehearsal therapy; REM, rapid eye movement.

Depending on the definition, dreaming occurs in almost every sleep stage. However, prototypical dreams—those that most people would label as such—principally occur during REM sleep. As a result, neuroimaging studies were initially focused on REM sleep to uncover the objective neural underpinnings that explain dreaming [229–234]. A canonical signature emerged (Fig 4A). First, both primary and higher-order visual regions became strongly active as the brain entered REM sleep, aligning with the vivid visual nature of dreams. Second, areas involved in motor functions such as motor and premotor cortices, the cerebellum, and the basal ganglia also became activated, consistent with the perception of first-person actioned movements. Third, limbic brain areas responsible for emotional processing, including the amygdala, hippocampal formation, and anterior cingulate cortices, display heightened activity, potentially accounting for the intense emotional tone dreams commonly take [235–237]. More interesting, however, were large regions of the brain that showed a converse decrease in activity during this otherwise highly active brain state of REM sleep, including the prefrontal cortex, the functions of which include volitional control and deliberative decision-making. Without knowing the experience of the individual, or even their state, should one look at this stereotypical pattern of brain activity and predict what subjective experience the individual was having, it would be a reasonable description of dreaming: perception of visual elements, motor action, emotionally laden, layered with autobiographical memories, yet disorganized, illogical, and without volitional control.

This neuroimaging signature was a key first step in answering a more fundamental question of whether other species dream. Of course, animals cannot provide verbal dream reports, making proof of the dream process difficult to ascertain for anyone other than humans. Evidence from the study of animal models of REM behavior disorder (RBD) provides added clues. Human RBD is a disorder characterized by dream enactment behaviors caused by the loss of muscle paralysis that accompanies the dreaming state of REM sleep [238]. Commonly violent, these movements may endanger the patient and their bed partners and, as recently affirmed, can reliably anticipate Parkinson’s disease [239]. Genetic, surgical, and pharmacological manipulation of the neural mechanisms that otherwise instigate muscle paralysis in rodents and cats results in remarkably similar behavioral repertoires during REM sleep, suggesting the possibility of dreams in animals (although there remains debate as to the acceptance of this premise [240,241]).

However, knowing the neural brain signature of human REM sleep that is associated with dreaming, scientists have started to ask whether a similar, objective neural signature could be found in other species and are getting closer to positive proof. The first fMRI-measured indications were recently published in *Columba livia* (pigeons). The researchers imaged the pigeon brains during the waking state and then again during REM sleep. What emerged was a strikingly similar pattern of brain activity to that associated with the experience of dreaming in humans; increased neural activity in visual regions, areas of the motor cortex, and subcortex, together with regions that regulate emotional affective states [242]. Of course, this finding does not prove that pigeons dream, but it does suggest that pigeons, despite their evolutionary divergence from mammals, nevertheless express a REM sleep neural pattern resembling that of humans, who do dream. Since neuroimaging methods in humans are fast becoming capable of deconstructing and then visually reconstructing the subjective waking experience of humans [243–245], a similar visual reconstruction of pigeon REM sleep brain activity may soon be on the horizon.

Not only does the brain show surges in stereotypical activity during human REM sleep, but so too do the peripheral nervous system, respiratory system, and vascular system [246]. A surprising recent discovery was made in cephalopods, specifically octopi, who have no central structured brain. During their offline state of sleep, the researchers systematically observed very clear, cycling swells of nervous system activity that repeated in bouts (approximately 1 minute) that are now believed to potentially be primitive REM sleep [247,248]. Also matching human REM sleep dreaming, it was harder to provoke the octopi to respond during these REM-like, fast-breathing, neural activation cycles relative to when awake [247]. Uniquely, these sleeping surges involved rapid pulsating body movements and synchronized changes in skin patterning. While this too does not prove dreaming, it is of note that octopi typically alter their skin patterning for camouflage during situations of threat and mating, both themes (threat and sex) that are present in human dreams [249,250].

### Why brains dream

The “why” of human dreaming has been one of the most contentious, and fiendishly difficult, questions to answer scientifically. Although subtle in distinction, to establish a function of dreaming, one has to determine that any such benefit is not simply that of the underlying biological sleep state from whence those dreams came (e.g., REM sleep) but is instead specific to the dream itself. Using this framework, 2 main functions of dreaming (above and beyond sleep or REM sleep) have so far emerged: associative memory and creativity, and emotional processing and mood recalibration.

#### Memory, association, and creativity

Although sleep is documented to boost learning and memory, only recently has dreaming been understood in terms of information processing independent of sleep. The benefits linked to dreaming are arguably even more powerful than the simple strengthening of individual facts that takes place during NREM sleep. Dreams can help interconnect large amounts of information, such that an individual wakes with a revised mind-wide web of associations capable of creatively divining solutions to problems previously faced while awake.

One recent study affirming this memory benefit assessed how efficiently individuals were able to weave together different memory components of a virtual maze they had been initially exposed to [251]. Those participants who obtained sleep after the learning phase were far better at assimilating the individual maze elements into a coherent whole, denoted by participants navigating their way through the maze faster, relative to a group that did not sleep during this time. This alone was not proof that dreaming itself was necessary. For that, researchers obtained dream reports throughout the sleep phase of those in the sleep group. Participants who slept and reported dreaming about the maze demonstrated a 10-fold improvement in navigation upon awakening, relative to those participants who slept, and still dreamt, but did not dream about the maze itself. It was not, therefore, enough to sleep or even to dream. Individuals had to dream about the waking problem itself in order to gain the associative memory benefit helping them to navigate the maze.

The process by which the dreaming brain accomplishes information assimilation, abstraction, and creativity is not completed in a single night. When studying dream content and waking life events systematically, information that individuals experience is most strongly integrated into their dreams over the first 2 nights, after which that information reprocessing appears to fade [252,253] (Fig 4B). Yet, these waking events then unexpectedly but very reliably resurface again 5 to 7 nights later—a phenomenon known as the “dream lag” effect [254]. These findings suggest that the conscious act of dreaming, and perhaps its memory function, evolves in 2 distinct temporal waves. The end product of these processes is arguably the difference between knowledge (learning individual facts, largely the role of NREM sleep [255]) and wisdom (knowing what they mean when we put them together, the role of conscious dreaming). Indeed, there is no shortage of science-related anecdotes of dream-instigated creativity. Examples include the dreams of Otto Loewi, which inspired experiments that led to his Nobel Prize–winning discovery of neurochemical transmission [256], and the equally impressive dream-inspired creative insights gifting Dmitri Mendeleev the elemental, universal (in the literal sense), conception of the periodic table of elements. Little wonder the advice is never to “stay awake on a problem.”

#### Emotional processing

Posttraumatic stress disorder (PTSD), which reflects an inability of the brain to process and ultimately overcome a mentally damaging event, epitomizes the disability that occurs when the brain’s otherwise normal ability to resolve and move past difficult, painful experiences becomes impaired. Reactive depression to a specific event, such as bereavement or divorce, offers another clinical example of challenging mental health resolution. Dreaming appears to be one mechanism through which such emotional restitution (or overnight therapy) is accomplished [175]. Although Sigmund Freud arguably opined some version of this dream benefit [257], it was seminal works by Rosalind Cartwright and her colleagues in the 1990s that provided initial, scientifically credible evidence. Cartwright studied individuals with reactive depression, assessing their sleep and dream content, and tracking their clinical progress over time. Patients with depression exhibit significantly fewer dream reports relative to controls [258,259], and the more severe the patient rated their depression, the fewer dream reports they mustered [260]. Yet, it was what the patients were dreaming about, more than simply if they dreamt, that predicted recovery. Patients who ultimately overcame their depression, accomplishing remission a year later, were dreaming expressly about the trigger of their depression (i.e., the content of their dream), relative to those who were dreaming, yet not about the inciting experience as much [261,262]. Adding to this evidence, recent data have confirmed that the negative and positive emotions nested within the dream content predict next-day waking mood changes [263], with negative dreams increasing negative mood and vice versa. Thus, more than just the state of REM sleep, indeed more than the act of dreaming, it seems that the content of one’s dreams, and their emotionality, offers a form of nocturnal emotional first aid [264].

Understanding how dreams are curated and used by the brain has led to the development of new dream therapies, such as imagery rehearsal therapy (IRT), specifically targeting the most distressing dreams common in nightmare disorder and prevalent in PTSD [265]. IRT is a cognitive-behavioral technique aimed at reducing nightmares by modifying the content of distressing dreams. Individuals learn to “rescript” and mentally rehearse revised versions of their nightmares while awake, which helps transform the narrative and reduce the frequency and intensity of distressing dreams (Fig 4C). For example, having been in a serious car accident, someone might say they have a terrible repeating nightmare where they are unable to steer their car out of the way of incoming traffic, the brakes stop working, and then BANG … they wake up utterly distraught. The therapist will ask them to imagine alternative endings to their nightmare (the imagery part). So, in our example, perhaps they now reimagine a different ending where they realize they can reach down and gently use the handbrake to slow the car down. Next is the rehearsal part, where patients would rehearse this less distressing alternate ending daily for a couple of weeks in the hope of modifying and updating nightmare memories. Indeed, in a recent study using IRT, patients with nightmare disorder experienced a significant reduction in their nightmare frequency [266].

However, the therapeutic potential of IRT was even greater. In a second group of patients, the researchers asked participants to rehearse their alternative endings while listening to a pleasing piano chord played every 10 seconds in the background. Then, over the ensuing 2 weeks of the study, that same chord was played to participants at sub-awakening volume whenever they entered REM sleep at night. The purpose was to trigger the memory of those rehearsed alternative endings when nightmares often manifest. This dual manipulation strategy resulted in an even greater reduction in nightmare frequency. Indeed, they experienced an 80% greater relative reduction in nightmare frequency compared with the group that received IRT alone. Moreover, these sound-paired participants also reported a 2-fold greater increase in the number of positive emotions in their dreams, relative to the control group. Even more remarkable, the added benefits of the combined IRT and sound protocol were still significant in a 3-month follow-up, despite the fact participants were no longer receiving any cues during the night. Such advances illuminate a path forward in altering dream content to better facilitate the innate “overnight therapy” of dreams long after awakening.

### Peering into dreams

Until recently, an individual’s dreams have been their own: a private experience that one decides if and when to reveal to others. Using advanced fMRI scanning methods, new data suggest this may no longer be the case [267]. While awake inside the MRI scanner, participants viewed many different objects across category themes (faces, cars, houses, etc.), with scientists then training a machine-learning model on this waking “ground truth” data of brain activity. The participants then performed a second fMRI scanning session. Now they were allowed to fall asleep, and the researchers obtained dream reports from these scanned sleeping periods. With high statistical probability, and using only the brain activity, blind to what the individuals had been dreaming of, the fMRI scans were able to predict what the individuals had experienced in their dreams with 70% accuracy. More specifically, the scientists could predict the category themes the individuals were dreaming about; e.g., they could decode that the dream included a car, predicting the form of the dream someone else was having. However, they could still not predict the unique content of the dreams (e.g., the make or model of the car), that level of detailed knowledge remains private—the purview of the dream owners themselves, at least for now. Based on recent EEG findings focused on specific regional brainwave frequencies, that time may be closer than once believed [268]. These developments could give rise to significant privacy concerns, considering that EEG is increasingly applicable to the home environment, and in the future, perhaps might even be as commonplace as contemporary smart wristbands [269].

### Lucidity

Although most people do not have volitional control over their dreams within dreams, some individuals do. Such volitional awareness of, and controlling choices and actions, is called lucid dreaming. The challenge has been to empirically prove such a seemingly unprovable assertion, considering that the lucid dreamer in question is asleep, and thus unable to communicate with scientists, paralyzed by the dream state. Yet, scientists have overcome this challenge by harnessing one of the few muscle groups spared from REM sleep paralysis; the extraocular eye muscles. It is, therefore, possible to train lucid dreamers in a pre-agreed-upon pattern of what is essentially eye movement Morse code. Using this, it enabled the participants to signal to scientists the moment when they gain lucid control of their dream and further signal what they claim to be doing as they were lucid dream [270].

One elegant experiment dispelling any doubt of lucidity combined this eye movement communication method with neuroimaging [271]. After signaling the initial state of lucidity when asleep inside the scanner, the participants provided a pre-agreed eye movement signal that they were about to deliberately clench their left hand in their dream, and another signal that they were then clenching their right hand. When compared to the waking “ground truth” brain activity of actual physical hand movement, the brain activity occurring during the claimed lucid dream hand movement was an unmistakable match. Thus, scientists obtained objective proof of the subjective claim of lucid dreaming.

Researchers have now gone further. Using these communication methods, they have had back-and-forth, real-time dialogues with lucid dreamers, in objectively convincing ways. After a participant indicated that they had gained lucidity, the experimenter posed simple mathematical questions to the dreamer (e.g., 8 minus 6) using either speakers or visual flashing codes that participants had previously learned [272]. The dreamer then responded using eye movements while still asleep in the lucid state (confirmed by EEG), providing their deliberated answer. The participants were able to respond correctly, with a level of accuracy far above chance. This finding offers further ratification of the lucid dreaming claim and indicates the ability of lucid dreamers to comprehend information in a volitionally conscious way, offering deliberate logical answers under willful control. While rudimentary, such evidence may open up the opportunity for sleep-dependent, and dream-dependent, interventions, boosting the innate benefits of dreaming to new realms.

## Lessons from the diversity of sleep across species

**Fig 5:**
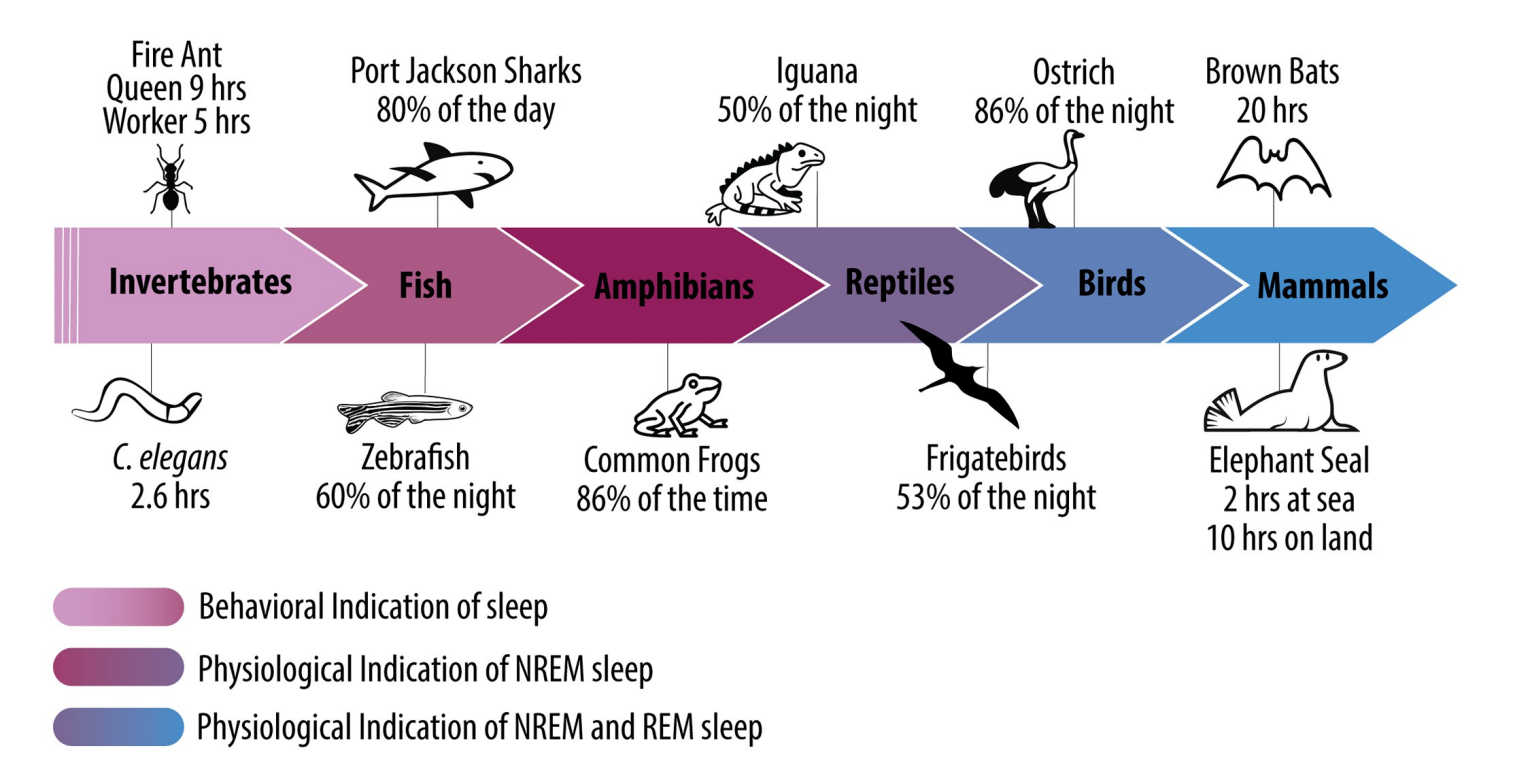
Sleep variability across species. Sleep duration varies markedly across the entire animal kingdom, from invertebrates to mammals. In most invertebrates and fish, sleep is defined behaviorally (e.g., for fire ants [276] and Port Jackson sharks [277]). Physiological evidence of NREM sleep can be found in a few amphibian species (e.g., the common frog [278]), as well as in reptiles [279]. In birds and mammals, evidence of sleep includes physiological recordings of both REM and NREM sleep, often measured in the lab [280–282]. NREM, nonrapid eye movement; REM, rapid eye movement.

Every organism that has been carefully studied to date sleeps. From vertebrates to boneless species such as jellyfish [273], octopuses [274], and even worms [275], sleep appears to be evolutionarily ancient, strongly conserved, and near universal. Yet, there is confusing controversy within this narrative of consistency. Being as strongly preserved across evolution as it is, one would assume that the amount of sleep, and how that sleep is structured, would similarly be consistent across species or, at the very least, more similar than different. The opposite is true: The only thing more axiomatic and surprising than the homogeneity of sleep across species is the heterogeneity of how sleep is expressed across and even within species. This is mirrored by the nearly equal numbers of hypotheses attempting to explain these differences. In this section, we outline long-standing evidence and several new discoveries seeking to decipher this perplexing heterogeneity (Fig 5).

### Sleep variability

In mammals, sleep duration can range from as little as 2 hours in elephants [283], to 20 hours in the little brown bat [282]—a 10-fold difference in sleep duration. Indeed, some [284] have pointed out how idiosyncratic sleep duration is by highlighting clever comparisons [284]. One example is the ground squirrel, which sleeps for 15.9 hours on average, while the degu, which is ranked within the same taxonomic order, sleeps for an average of just 7.7 hours. By contrast, animals from different taxonomic orders, such as the guinea pig and the baboon, sleep for an identical amount of time (9.4 hours).

Recent data have added a new dimension of sleep variability within the same species. Elephant seals sleep for a little over 2 hours when making their months-long trips at sea, yet once they return to the land, they will defy that trend and consistently sleep for 10 hours or more each day (Fig 5) [285]. Similarly, male pectoral sandpipers reduce their sleep amount 2-fold when females are present and fertile, relative to their sleep during nonbreeding periods. This adaptive act of ecologically pressured sleep reduction comes with a benefit. Those birds that slept the least during the breeding period gave rise to the most offspring [286]. Therefore, just when a fixed sleep duration label for a given species had been assumed from short-term, in-laboratory evaluations, it turned out that sleep duration within the same animal is far from fixed when tracked longitudinally in the wild.

Beyond sleep duration, species also differ in how their brains obtain sleep. During their trans-oceanic migrations, birds can switch off an entire hemisphere of their brain, putting it into a deep sleep while the other half of the brain remains wide awake. This feature, termed “unihemispheric sleep,” allows the migrating birds to have an open eye (linked to the respective waking hemisphere) turned to face the direction of flight [280]. The other eye, connected to the sleeping hemisphere, is closed shut. Ducks use this same sleep adaptation in response to a different evolutionary pressure. When ducks are lined up in a row, those individuals at the far ends of the flock sleep unihemispherically, directing the open waking eye to the vulnerable side of the flock to monitor predatory threats, while the eye (and corresponding opposite hemisphere) facing inwards to the safety of the flock is closed due to sleep. Those ducks seated within the flock have the luxury of sleeping with both hemispheres, since 360° perimeter threat detection is covered by the 2 sentinel ducks sitting at each end of the flock [287]. Many aquatic mammals show similar unihemispheric sleep patterns, driven by the equally different evolutionary pressure of needing to continue surface breathing. Nevertheless, they still manage to accomplish their sleep needs without drowning, one-half of the brain at a time.

Satisfying one’s full sleep need while keeping one-half of the brain awake to continue waking activities would seem like an envious ability that humans do not possess. Or so it was thought. Recent reports have shown that humans do, in fact, perform a version of unihemispheric sleep, albeit one that does not afford us the ability to continue functioning as if one hemisphere is completely awake. Have you ever had the experience of feeling as though you did not sleep well in a strange new environment [288]? A study has shown that when individuals sleep in a new environment, one hemisphere of the brain sleeps in a more shallow state of deep NREM sleep than the other. As with the ducks, the interpretation is that the human brain adapted to a change in how it sleeps when the potential for threat increases. While not fully awake, half of the brain in the proposed threat detection mode of lighter sleep is more responsive to sensory stimuli [289].

### Theories of sleep variability

Building on these findings, new theories have been set forth regarding the explanatory functions of how sleep developed across the tree of life. Specifically, since REM sleep appears to be exclusive to warm-blooded animals, it has been proposed that thermoregulation was the original evolutionary force behind the development of REM sleep as a novel state designed to maintain homeothermy while still accomplishing sleep [290,291]. Consistent with this view, brain temperature drops in homeotherms (including humans) as they go deeper into NREM sleep. However, this trend reverses during REM sleep, when brain temperature reliably increases. Thus, REM sleep is thought to have emerged due to the evolutionary need for a reheat cycle during sleep; otherwise, the cognitively slowed state of brain functioning and the impeded autonomic function often necessary upon waking up quickly would be unacceptable for survival and fitness. Thus, the evolutionary reason that REM sleep emerged in warm-blooded mammals and birds (the classes that show reliable REM sleep) was to protect against excessive central brain hypothermia that would otherwise occur by experiencing NREM sleep alone [291]. However, the recent discovery of a “proto” version of REM sleep in cold-blooded reptiles [279,292,293], zebrafish [294], and marine invertebrates (including cuttlefish and octopuses [247,248]) has challenged the REM sleep thermoregulation hypothesis. Instead, these new findings suggest that the features of REM sleep, from muscle paralysis to rapid eye movements and increased cortical activity, developed earlier than previously thought. Therefore, the evolutionary reason for developing REM sleep must have preceded the need for temperature regulation. As with all good new discoveries, these data have only led to more interesting, as yet unanswered, questions about the function of REM sleep. The initial evolutionary reason for the emergence of paradoxical sleep, or REM sleep, therefore, still remains a paradox.

Even theories that sought to explain the variability in sleep duration across species, above and beyond the individual stages, remain controversial. A prominent and simple first theory was that brain size is the explanatory factor. The brain is, after all, a disproportionately demanding metabolic organ, and this size-related cost may, therefore, explain differences in sleep amount. Not so. The variability in sleep amount across species is not explained by brain size nor is it explained by cognitive ability [295]. Modified theories focused next on metabolic rate. One may logically predict that the more metabolically active a species, the more sleep (i.e., energy savings) it requires. Yet, metabolic rate only marginally accounts for variability in sleep amount, and even there, the association is in the opposite direction. Species with a high metabolic rate sleep less than those with a lower rate [284]. The current explanation is that more metabolically active species must spend more time awake foraging for food to satisfy their greater caloric demand and, thus, sleep less [2,291]. Here, too, as in the case of the flocking ducks, predatory risk further shapes sleep variability. Analysis of sleep duration across 58 species of mammals indicated that indeed those exposed to higher predatory risk sleep less, even when correcting for the size of the animal [295]. But since predation risk and being a herbivore tend to correlate, which factor is the more important is still an open question. One especially interesting species is the omnivore baboon (*Papio anubis*), which is subject to nightly predation risk imposed by leopards and lions. To mitigate the risk, and similar to the ducks, the baboons sleep in groups and also alternate sleep locations, 2 modifications that tend to compromise their sleep amount due to, what seems like, the first night effect (described for humans above) and awakenings caused by fellow baboons [296]. Despite the survival benefit of sleeping close to conspecifics, the price, in the form of insufficient sleep, has been observed across many mammals sleeping in the wild [295].

These are just some of the many examples of theories seeking to explain the heterogeneity of sleep among species. What all of these examples (from unihemispheric sleep to a 10-fold difference in sleep needs) have taught us is perhaps obvious, but worth reiteration: If sleep were dispensable and, thus, optional, or even if certain stages of sleep were desirable but not required, evolutionary pressures would have led to sleep, or a specific stage of sleep, being forfeited a long time ago. Nevertheless, the fact that sleep has consistently persevered throughout the evolution process, and done so in ingenious ways (within an individual, and across groups of individuals), serves to reinforce the conclusion that sleep is a critical necessity, serving numerous functions within and across species. This may not be so surprising, considering we have long recognized the polyfunctional nature of wakefulness.

## Conclusions

This collection of recent discoveries not only affirms the role of sleep as a biological life-sustaining necessity but also extends the polyfunctional nature of sleep and the conscious state of dreaming in unexpected ways. These include DNA repair, immune function governance, effects on the gut microbiota, brain cleansing, controlling and enhancing complex social and emotional functioning, novel means of memory optimization, and the co-opting of divergent creativity. Moreover, through a growing understanding of basic sleep physiology, mechanism, and function, a plethora of new technologies are emerging that are capable of manipulating and enhancing human sleep physiology. As a result, there is a distinct possibility in the future that humans will be able to therapeutically manipulate sleep in precise ways for the treatment of specific diseases and disorders. These may range from regulating the gut microbiota to managing the mental health of an individual, slowing brain aging and its pathologies, aiding in trauma resolution, and even facilitating prosocial engagement in the face of a growing loneliness epidemic [297–299].

Apart from this, a different discussion theme emerges from the new wave of research discoveries, i.e., the elemental “why” of sleep, and more specifically, how researchers conceptualize the question, aside from any answers they arrive at. Wakefulness, the antithesis of sleep, becomes a meaningful lens through which to explore a claimed sleep-dependent benefit/process. In this framework of questioning, the guiding principle has been to search for a sleep function that cannot be served by wakeful rest. The query, therefore, becomes, for any functions thought to be ascribed to sleep, is there any evidence that this function can be supported by wakefulness, and if not, why not?

Another nonmutually exclusive framework for answering the question of sleep’s why is in its absence. Classical methods of total and selective sleep deprivation were scientifically limited based on the confounds of the deprivation methods used, such as stress, or the fact that selective deprivation of a sleep stage also meant that total sleep time was defacto reduced. Now, however, there are much more sophisticated methods that obviate many of these concerns and offer stronger causal affirmations. A good example is the method of sub-awakening auditory tones. Using this method, the individual is selectively deprived of deep NREM sleep in a specific brain area by the tones that lift them into lighter NREM sleep without waking them, and so total NREM sleep duration is preserved [300]. Another example is using implanted electrodes in animal models. Here, the electrodes are used to selectively disrupt neural events, such as forward memory-sequence replay during NREM sleep (the replaying of the order of memory-cell firing that was coded during initial spatial learning while the animal was awake), thereby demonstrating a causal dependence on a physiological sleep mechanism for memory consolidation [301]. As new methodological advances grow in their nuance and ability to selectively excise other stages of sleep, or even specific electrical brain-wave oscillations, the dissection of the “why” of sleep dependency will become ever more concrete. And “dependent” not only in the sense of sleep versus wake, but also of one sleep state relative to another, or even to the extent of one specific brain region’s experiences of a sleep-oscillation state relative to other brain regions.

More generally, the revelations brought forth by these new, highly diverse functions of sleep do not negate the possibility that one consensus and common function of sleep nevertheless exists across species. There may very well be a singular (original and/or common) function of sleep that transcends taxonomy. Moreover, the notions that a single common function of sleep exists, while additional multiple functions of sleep have later evolved across time, are not mutually exclusive or antagonistic.

As the polyfunctional view of sleep grows, another fruitful framework is that of the interdependence and interconnectedness of different sleep functions that achieve benefits to the organism greater than the sum of each part. Here again, it is something that has long been accepted regarding many of the functions of wakefulness. For example, without sleep’s interconnected support, the ensuing free radical damage caused by sleep deficiency may increase inflammation, which, in turn, leads to sickness behavior, which consequently triggers social withdrawal and loneliness in the sleep-deprived individual. Another possible example would be the emotional and social changes in behavior caused by sleep loss that impair the immune system, which leads to worse gut microbiome health and, through afferent vagal signaling, alters mood and emotional states, each of which only further disrupts sleep, leading to an interconnected negative spiral. For sleep and its functions, cinematically speaking, it is “Everything, Everywhere, All At Once.”

## Abbreviations

ADHD: attention deficit hyperactivity disorder
ASD: autism spectrum disorder
CSF: cerebrospinal fluid
DORA: dual orexin receptor antagonist
EEG: electroencephalogram
fMRI: functional MRI
HPA: hypothalamic-pituitary-adrenal
IRT: imagery rehearsal therapy
NREM: nonrapid eye movement
PTSD: posttraumatic stress disorder
RBD: REM behavior disorder
REM: rapid eye movement
SWA: slow-wave activity
